# Electron ptychography reveals correlated lattice vibrations at atomic resolution

**DOI:** 10.1038/s41467-026-74135-4

**Published:** 2026-06-10

**Authors:** Anton Gladyshev, Benedikt Haas, Thomas C. Pekin, Tara M. Boland, Marcel Schloz, Peter Rez, Christoph T. Koch

**Affiliations:** 1 Department of Physics, Humboldt-Universität zu Berlin & Center for the Science of Materials Berlin, Berlin, Germany; 2 School for Engineering of Matter Transport and Energy, Arizona State University, Tempe, AZ USA; 3 Computational Atomic-Scale Materials Design (CAMD), Technical University of Denmark, Kgs. Lyngby, Denmark; 4 Department of Physics, Arizona State University, Tempe, AZ USA

**Keywords:** Transmission electron microscopy, Imaging techniques, Computational methods, Two-dimensional materials

## Abstract

Electron Ptychography is a computational imaging technique capable of performing phase retrieval at atomic resolution. Here we introduce the CAVIAR framework (Correlated Atomic Vibration Imaging with sub-Angstrom Resolution) that reveals spatial correlations in atomic displacements at the atomic scale. Using realistically simulated data for a symmetric *Σ*9 grain boundary in silicon and experimental data of a hexagonal boron nitride bicrystal, we observe correlations between atomic movements in the range of 10-20 pm at room temperature in agreement with our expectation. From only the atomic masses and temperature as input, we obtain frequencies of the longitudinal and transverse acoustic and optic phonons from just a few nm^3^ volume, in agreement with inelastic neutron scattering. This ability to spatially resolve correlated atomic motion distinguishes CAVIAR and positions it as a complementary tool to vibrational electron energy loss spectroscopy for exploring atom dynamics at the finest scale.

## Abstract (web summary)

The authors present a method that extends conventional electron ptychography to reveal spatial correlations in atom displacements at the atomic scale. These correlations can be used to directly analyse the response of defects in crystals to external perturbations or to construct the dynamical matrix for the crystal, yielding its phonon dispersion.

## Introduction

While capable of imaging the lateral positions of atomic columns constituting thin slabs of material, the achievable resolution of conventional electron imaging techniques in a transmission electron microscope (TEM) is very sensitive to instrumental imperfections: the partial coherence of the electron source, lens aberrations as well as mechanical and electronic instabilities of the microscope. Ptychography^1–5^ is a computational phase retrieval technique that, to some extent, can compensate for the imperfections of the equipment. Numerous quite different variations of this technique exist, e.g., Fourier and near-field ptychography^6,7^, and a variety of reconstruction schemes for them. The method used in this paper, “classical” far-field ptychography, recovers a complex transmission function of a specimen from a four-dimensional scanning transmission electron microscopy (4D-STEM) dataset containing transmitted intensities^8^ collected with a pixelated detector while illuminating overlapping areas of its surface with a convergent beam.

**Fig. 1:**
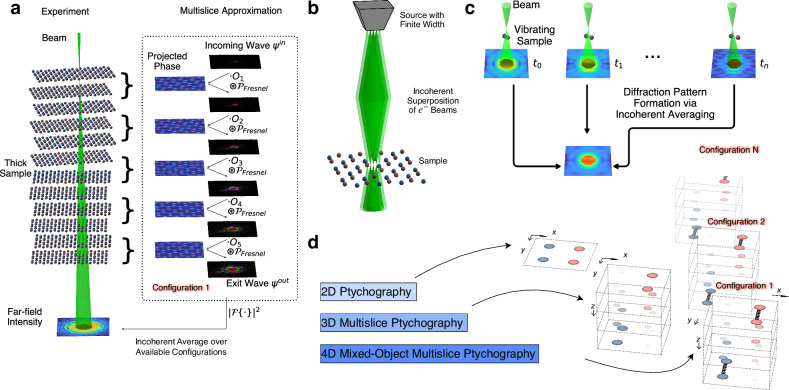
Principles of numerical diffraction pattern formation in ptychography. **a** Schematic illustration of the multislice 4D-STEM simulation for a thick sample. First, the beam propagation is split into multiple intervals (5 in the depicted case). The projected electrostatic potential from each of the intervals (slices) constitutes the phase of the corresponding transmission function *O*_*i*_. The exit wavefront *ψ*^*out*^ is obtained via sequential application of the transmission functions *O*_*i*_ and Fresnel propagators ${{{\mathcal{P}}}}_{Fresnel}$ to the incoming wavefront *ψ*^*in*^^55^. The diffraction pattern is calculated as the squared modulus of the Fourier transformed exit wave. In order to account for various types of incoherent scattering, the diffraction pattern is obtained as the sum over the scattered intensities from different configurations in the detector plane. Two types of variations in configuration are depicted in (**b**, **c**). **b** The effect of finite electron source size, creating a superposed ensemble of shifted beams where the occurrence probability of each beam is approximated to obey a Gaussian distribution. **c**, Principle of thermal diffuse scattering (TDS) caused by atomic movement. The diffraction patterns corresponding to quasi-static sample configurations^14,17,35^ at multiple points in time *t*_*i*_ are incoherently summed to obtain an expected intensity of a diffraction pattern with diffuse background. **d** Electron ptychography reconstruction models with increasing complexity levels: 2D projected phase reconstruction, 3D multislice ptychography and our proposed reconstruction method—4D mixed-object multislice ptychography, where the sequence of object states can be transformed to obtain correlations in variations of interatomic distances, schematically depicted as springs.

Ptychography has proven itself as a powerful tool for imaging of thin samples^5,9^. Recently, it was shown that adopting a multislice formalism makes it possible to resolve specimen features as fine as the blurring due to the vibrations of atoms^10^, an achievement that triggered a rapid rise in popularity of electron ptychography in the community. It was demonstrated that spatially non-uniform vibrational modes can be fitted to the atomic shapes produced via ptychography^11^. However, fully surpassing this limit of atomic vibrations was not possible, since a coherent specimen model as depicted in Fig. 1a cannot describe the formation of an incoherent diffraction pattern of the type schematically shown in Fig. 1c. Previously, there were attempts to include an incoherent sample model in experiments with laser^12^ and X-ray illumination^13,14^, but applications to electron ptychography assumed only uncorrelated atomic vibrations^15,16^.

Here we propose to utilize thermal diffuse scattering (TDS)^17^ as a source of information about correlated atomic movement rather than the ultimate limiting factor in achievable spatial resolution^10^. This information can be retrieved using the ptychographic reconstruction scheme CAVIAR (Correlated Atomic Vibration Imaging with sub-Å ngstrom Resolution), presented here, which is based on mixed-object electron ptychography. It combines a mixed-object formalism^14^, where the specimen is modeled as a statistical ensemble of states, with lattice Green’s function analysis^18^, a technique well-established in molecular dynamics (MD) for quantifying vibrational correlations. The evolution from 2D ptychography^19^ to 3D multislice ptychography^10,20^ to CAVIAR is shown in Fig. 1d. With the retrieval of information about correlated movements of atoms (at atomic resolution), mixed-object ptychography becomes a complementary technique to vibrational electron energy-loss spectroscopy (EELS) in scanning transmission electron microscopy (STEM)^21,22^. Vibrational STEM-EELS has been very successful in studying vibrational phenomena down to the atomic scale, such as vibrational states of single atoms^23^ or localized modes at grain boundaries^24^. Vibrational EELS has an inherently limited energy resolution and lower energy limit it can probe. It can either be employed at atomic resolution without substantial momentum-resolution or momentum-resolved for mapping phonon dispersions but then with a spatial resolution insufficient for resolving atoms, as implied by Heisenberg’s uncertainty principle. As mixed-object ptychography works fundamentally different from EELS, it offers complementary information about the correlated motion of neighboring atoms at atomic resolution and should prove useful as another tool for the study of atomic vibrations at the finest scale. As both techniques are based on STEM, they can, in principle, be performed using the same instrument, given the necessary hardware.

## Results

The first step of CAVIAR is a gradient-based ptychographic reconstruction^5,10,20,25–28^ which fits a transmission function of the specimen by determining the most likely^29^ parameters of a numerical model for a given set of experimentally measured intensities. Various aspects of the numerical model are depicted in Fig. 1 and a detailed mathematical description can be found in the Supplementary Eqs. 1–7. Upon convergence of the fit, one obtains a discrete complex transmission function of the specimen, which in our case has four dimensions: two lateral coordinates (*x*,*y*) spanning a plane perpendicular to the beam direction, an out-of-plane coordinate (*z*), parallel to the beam; and one dimension accommodating incoherent object modes (*n*). The phase of the transmission function *O*(*x*, *y*, *z*, *n*) is directly proportional to the projected electrostatic potential in slice *z*^10^ of the *n*’th configuration of the object. The second step of CAVIAR is an extraction of the interatomic correlations from an unordered sequence of the retrieved object states *n*. To do so, we adopt a framework developed for the calculation of phonon dispersion curves from MD simulations^18^. The initial determination of atomic positions can be achieved either manually or, in more complex scenarios, using an object detection network (e.g., ref. ^30^). Subsequently, refining the atomic coordinates can be accomplished by calculating the center of mass within a small circular region around the initially determined atomic positions, using the retrieved phase values as weighting factors. In this way, for each unit cell *l*, basis atom *κ*, direction of displacement *α* (*x*,*y*,*z*) and reconstructed object state *n* one can get real-space atomic positions *R*_*lκαn*_ and displacements from the mean position^18^:

1 $${u}_{l\kappa \alpha n}={R}_{l\kappa \alpha n}-\frac{1}{N}{\sum }_{n=1}^{N}{R}_{l\kappa \alpha n}.$$

Afterwards, the lattice Green’s function coefficient ${G}_{l\kappa \alpha {l}^{{\prime} }{\kappa }^{{\prime} }{\alpha }^{{\prime} }}$ describing the correlations can be computed as a second moment of the displacements^18,31^:

2 $${G}_{l\kappa \alpha {l}^{{\prime} }{\kappa }^{{\prime} }{\alpha }^{{\prime} }}=\frac{1}{N}{\sum }_{n=1}^{N}{u}_{l\kappa \alpha n}{u}_{{l}^{{\prime} }{\kappa }^{{\prime} }{\alpha }^{{\prime} }n}.$$

### Simulated data

To investigate the feasibility of CAVIAR, we started with simulated data to have well-controlled conditions and the ability to compare with the ground truth, before considering experimental data. As a test sample, we chose a symmetric *Σ*9 grain boundary in silicon, MD simulations of phonon spectra of which^32^ agree perfectly well with experimental STEM-EELS data^24^. Moreover, prior work has shown that atoms at the grain boundary exhibit distinct vibrational behavior compared to those in bulk regions due to differences in bonding environments^24^. CAVIAR enables this phenomenon to be examined at the level of individual atoms.

**Fig. 2:**
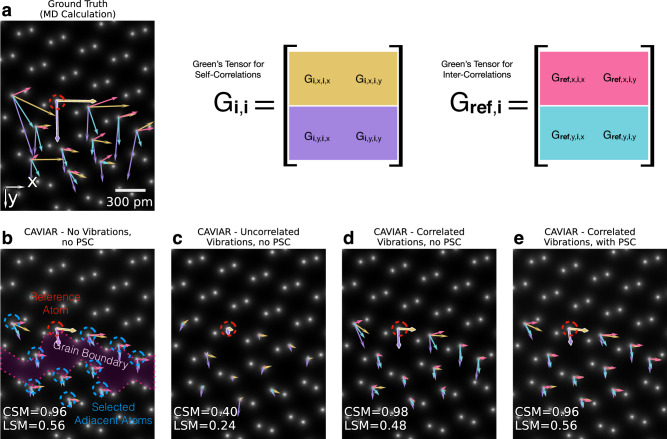
Ptychographic reconstructions from realistically simulated data for various kinds of atomic motion and partial spatial coherence (PSC). **a** Ground truth phase image from an MD simulation of a *Σ*9 grain boundary in silicon with arrows indicating various components of the Green’s tensor defined in eq. (2). A randomly picked reference atom is marked by a red circle in (**a**–**e**), its selected neighbors and the grain boundary are shown by a blue and magenta dashed lines in (**b**), respectively. Colors of arrows show different correlation types: yellow/purple (self-correlations), pink/light blue (correlation with reference atom), two 2 × 2 blocks of the full Green’s tensor are shown schematically in the middle of the top row using the same colors. **b**–**e** Reconstructed phases and correlation matrices obtained from data simulated using different levels of approximation: **b** Perfectly coherent dataset, no PSC—no correlations appear to have been reconstructed; **c** Uncorrelated vibrations (Einstein model), no PSC—correlations between neighbors largely vanish; **d** Correlated vibrations, no PSC—principal directions of correlations match the ground truth; **e** Correlated vibrations, with PSC—the recovered correlations match the ground truth and the offset created by PSC is efficiently mitigated by multiple probe modes; In the bottom of images **b**–**e** we report values of length-similarity (LSM) and cosine-similarity (CSM) metrics (cf. eqs. (3) and (4)) between MD ground truth and retrieved correlation vectors. All reconstructions used 4 object slices, 20 object states. Reconstructions in (**b**–**d)** were done with a single probe mode. In the reconstruction (**e**) 9 probe modes were used.

To generate a 4D-STEM dataset^33^ we used 30 time-snapshots (object configurations) of a 0.7 nm thick MD super-cell containing four atomic layers. The accelerating voltage, convergence semi-angle, and scan-step were 200 kV, 30 mrad, and 50 pm, respectively. Averaging neighboring diffraction patterns with a Gaussian weighting was used to simulate the effect of partial spatial coherence with an effective source size (full width at half maximum of the Gaussian) of 47 pm. In total we generated four 4D-STEM datasets mimicking different degrees of incoherent scattering: (1) a perfectly coherent dataset based on a single MD snapshot, i.e., without TDS and without additional partial spatial coherence; (2) a dataset based on the Einstein model of thermal vibration^17,34,35^, i.e., uncorrelated displacements sampled from a normal distribution with standard deviation matched to the average one from the MD data and without additional partial spatial coherence; (3) a dataset based on MD data including correlated atomic movement without partial spatial coherence; (4) a dataset based on MD data with additional partial spatial coherence. Allowing for partial spatial coherence in the 4D-STEM data is important due to the fact that averaging over multiple slightly displaced probes is conceptually equivalent to averaging over multiple slightly displaced objects. Thus, when the single probe mode is used in a reconstruction, partial spatial coherence, absorbed by the object states, introduces a positive offset to the *x*-*x* and *y*-*y* correlations of the atomic coordinates. The full Green’s tensors in Supplementary Fig. 2 reflect this effect, showing a uniform offset in the *x*-*x* and *y*-*y* correlations in the single probe mode reconstructions from the datasets corrupted by partial spatial coherence. Mitigation of this effect can be achieved in two ways: (1) the positive offset can be corrected in the recovered correlations, for example, in the same manner as background subtraction is performed in EELS spectra^24^; (2) A ptychographic fit with single object mode and multiple probe modes^10,14^ can be performed prior to mixed-object and mixed-probe reconstruction in order to fit multiple probe states that will absorb the offset. In the reconstructions presented further we chose the second approach. All ptychographic fits from the Si grain boundary data were performed using 20 object states and 4 slices with a spacing of *Δz* = 0.19 nm. In Fig. 2 we show both the reconstructed projected phase as well as the retrieved interatomic correlations. The reconstructions from data without additional partial spatial coherence were done with a single probe mode and the reconstruction in Fig. 2e was done with 9 probe modes.

Although there is no fundamental bound prohibiting a full 3D reconstruction, the *z*-resolution currently achievable with STEM-ptychography^20^ is not sufficient to quantify the out-of-plane vibrations. Therefore, we limit our analysis to the in-plane components of vibrations. For a system of *N* atoms and two spatial coordinates, the retrieved Green’s tensor depicting all correlations is a 2*N* × 2*N* matrix. In order to visualize it in a convenient form, we first randomly picked a reference atom, shown in Fig. 2b by the red circle, and some adjacent atoms circumscribed by the blue circles in the same image (for clarity of the figure only a few atoms were chosen). For a given pair of atoms we depict correlations as two vectors: the first one (coral) representing correlations of the reference atom’s *x* coordinate with the neighbor’s *x* and *y* coordinates and the second one (cyan) representing correlations between the reference atom’s *y* coordinate with its neighbor’s *x* and *y* coordinates. The yellow and purple arrows represent the atom’s auto-correlation, which corresponds to the two contributions $\langle {u}_{x}^{2}\rangle$ and $\langle {u}_{y}^{2}\rangle$ of the equivalent isotropic displacement factor^36^.

Figure 2a shows the ground truth of the displacement correlations from the MD snapshots. One can see that the correlations retrieved via CAVIAR (Fig. 2d, e) are weaker than they should be, but the angles between the depicted arrows are nearly identical to the ground truth. Further, with our approach we can distinguish between various kinds of vibrations. In case of a coherent simulation (Fig. 2b) we cannot detect any displacements at all and for the simulation with uncorrelated atom displacements (Einstein model, Fig. 2c) we do not recover any correlations between neighbors.

To quantitatively compare the correlations from the initial MD data and those extracted from the ptychographic reconstructions, we adopt two orthogonal metrics judging agreement in strength and direction: length similarity metric (LSM) and cosine-similarity metric (CSM)^37^. For each atomic pair *i*, whose correlations are represented in Fig. 2 by a vector *V*_*i*_ with two components (denoted *j*), we evaluate the similarity between the reconstructed (*Rec*) and ground truth (*GT*) correlations as follows:

3 $$\,{\mbox{LSM}}\,={\sum }_{ij}\sqrt{\frac{| {V}_{ij}^{Rec}{| }^{2}}{| {V}_{ij}^{GT}{| }^{2}}}$$

4 $$\,{\mbox{CSM}}\,=\frac{1}{{N}_{vectors}}{\sum }_{i=1}^{{N}_{vectors}}\frac{{\sum }_{j}{V}_{ij}^{GT}\cdot {V}_{ij}^{Rec}}{\sqrt{{\sum }_{j}{\left({V}_{ij}^{GT}\right)}^{2}}\cdot \sqrt{{\sum }_{j}{\left({V}_{ij}^{Rec}\right)}^{2}}}.$$

The CSM metric is bounded between −1 and 1, where 1 means perfect similarity. The LSM metric is bounded from below by zero, meaning the absence of vibrations in a reconstruction. LSM = 1 also means a perfect similarity. For the reconstruction from the dataset without partial spatial coherence (Fig. 2d) the CSM value is the highest (0.98), showing an almost perfect match. When adding partial spatial coherence (Fig. 2e), the mentioned positive offset in correlations reduces the CSM value only slightly to 0.96, indicating that multiple probe modes efficiently eliminate the ambiguity. For the coherent dataset and the one with uncorrelated TDS presented in Fig. 2b, c, the CSM values are substantially lower. The LSM metric allows to quantify the magnitude of retrieved vibrations, for a perfectly coherent dataset in Fig. 2b the value is close to zero, indicating the absence of atomic vibrations. Despite a good match in directions of correlations retrieved from correlated datasets, their magnitudes are approximately two times lower than the ground truth. Overall, we conclude that we may underestimate the strength of correlations due to the resolution in these reconstructions being limited, as well as due to a limited number of object states, but we can explore the directionality and relative differences between neighboring bonds.

### Experimental data

After validating CAVIAR on simulated data we moved to an experimental 4D-STEM dataset of an approximately 15 nm thick hexagonal boron nitride (hBN) sample in [0001] direction, whose upper and lower halves were twisted by approximately 11^∘^ relative to each other around said zone axis^28^. The 4D-STEM dataset was acquired using a Nion HERMES microscope, operated at an accelerating voltage of 60 kV, with a convergence semi-angle of 40 mrad and a scan step of 30 pm. The reconstructed transmission function included 10 object states and 30 slices with a spacing of 0.48 nm. We further used 5 incoherent probe modes to account for partial spatial coherence of the beam and potentially occurring sample drift.

**Fig. 3:**
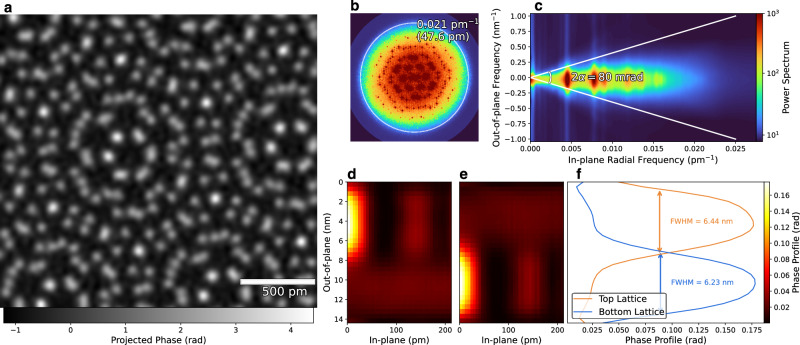
Experimental ptychographic reconstruction from a 4D-STEM dataset of an approximately 15 nm thick hBN bicrystal. The specimens upper- and lower-halves (the beam propagates vertically) are twisted with respect to each other by approximately 11^∘^. The reconstruction was carried out using 30 slices, 10 object states and 5 probe modes to account for partial spatial coherence and sample drift. **a** Reconstructed phase projected over all slices and averaged over all states. **b** Sum of moduli squared of the Fast Fourier-transforms of all slices and object states indicating an information transfer up to 47.8 pm, way above the double-aperture radius limit (61 pm for an accelerating voltage of 60 kV and convergence semi-angle of 40 mrad). **c** The azimuthal average of the 3D-Fourier moduli squared makes it possible to estimate the achieved resolution along the *z*-axis—approximately 2 nm for the first two rings of Bragg peaks. **d**, **e** Phase profiles averaged over all reconstructed atoms obtained by azimuthally averaging phase profiles around the centers of atomic columns within the upper and lower slices of the reconstruction, respectively. **f** Phase profiles along *z* through the centers of atomic columns clearly validating that two crystals have identical thicknesses within the precision limited by the depth resolution.

In Supplementary Fig. 1, we demonstrate that even for a thin *Σ*9 grain boundary in silicon, multiple scattering dominates over incoherence arising from sample vibrations. Consequently, successful extraction of interatomic correlations via CAVIAR requires both sufficient slice sampling and adequate depth resolution. To validate that these conditions were met in our experiments, we begin by analyzing the achieved spatial resolution. Figure 3a shows the projected phase, summed over all 30 *z*-slices. In Fig. 3b we show the power spectrum incoherently averaged over object slices and states, which indicates an information transfer up to *d* = 47.6 pm. Dividing the achieved resolution by the illumination wavelength *λ* = 4.87 pm gives us a value of *d* = 9.78*λ*. A ratio *d*/*λ* < 10 for a three dimensional potential reconstruction has so far been difficult to achieve^10^. In order to estimate the depth resolution we azimuthally averaged the 3D Fourier transform of the phase over the in-plane spatial frequencies. The corresponding power spectrum is shown in Fig. 3c and clearly indicates a missing wedge of three dimensional information^10^. Albeit the in-plane frequencies show an information transfer reaching beyond the double aperture limit, the depth spatial frequencies are still confined in a full convergence angle, in agreement with previously published multislice ptychographic reconstructions^20^. The first two rings of hBN reflections correspond to a depth resolution of approximately 2 nm, but at high scattering angles, the first-order Laue zone diffraction ring is reconstructed, indicating a vertical resolution better than 6.7 Å (see Supplementary Fig. 9). In order to illustrate the separation between upper and lower lattices of the sample we computed average phase profiles as a function of distance *r* to the center of the atomic columns in each of the reconstructed potential slices. In Fig. 3d, e these *r* − *z* phase maps for the upper and lower lattices are shown. In Fig. 3f the phase value at the center of the atomic columns is shown as a function of *z* (line profiles along the left hand sides of Fig. 3d, e). The boundaries of each sub-lattice as well as an overlap region caused by the limited resolution along the *z*-axis can clearly be identified.

**Fig. 4:**
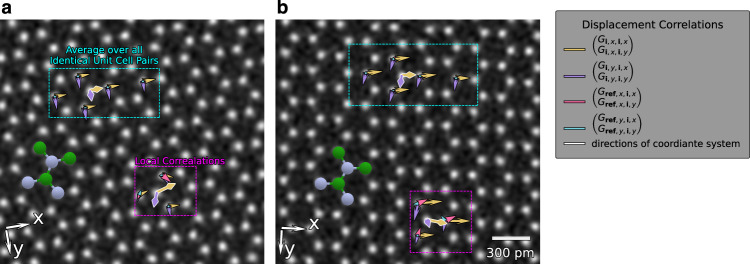
Interatomic correlations recovered from experimental mixed-object ptychographic reconstruction of approximately 15 nm thick bulk hBN crystal. **a**, **b** Projected phases of the reconstructed transmission function summed over all object states and 8 out of 30 slices selected from the *z*-ranges *z* = 1.9 nm …5.7 nm and *z* = 8.6 nm …12.5 nm, respectively. For each of the potential slices within those ranges the interatomic correlations were computed separately; their averages were computed afterwards. The depicted arrows represent various components of retrieved Green’s tensors. Randomly picked reference atoms are shown by white outline, yellow and purple arrows show correlations of atoms with their own displacement along *x*- and *y*-axes. Pink and light blue show correlations of atoms with displacements of a reference atom along *x* or *y* direction. Note that for the two stacked crystals the coordinate system was rotated to align the *x*-direction with one of the three nearest neighbors. The *y*-axis was then defined to be orthogonal to it. Directions of each coordinate system are shown with white arrows in the lower left corners of each panel as well as the unit cells. It is important to mention that individual atoms may vibrate quite differently, but on average they are expected to behave similarly. In the magenta colored dashed boxes we show correlations corresponding to a particular atomic column (and its neighbors) and in turquoise boxes we show correlations averaged over all identical unit-cell pairs (i.e., with the same differences $l-{l}^{{\prime} }$ and $\kappa -{\kappa }^{{\prime} }$). Note that principal directions of averaged correlations are identical in (**a**, **b**).

The depth resolution analysis above confirms that multiple scattering effects are accounted for in the reconstruction, enabling further analysis of interatomic correlations. For each of the 30 slices and 10 states we extracted atom positions and corresponding displacements. In Fig. 4a, b projected phases and interatomic correlations averaged in *z*-regions from 1.9 nm up to 5.7 nm and from 8.6 nm up to 12.5 nm, respectively, are shown. For each of the two lattices we evaluated correlations in coordinate systems aligned with the respective local lattice frame. In the magenta dashed boxes we show correlations corresponding to a particular atom column (and its neighbors) and in the turquoise boxes we show correlations averaged over all identical unit-cell pairs. The correlation vectors, overlaid at atomic sites, reveal similar spatial structures and orientations in both layers, despite their relative rotation. This confirms that the recovered correlations are physically meaningful and lattice-intrinsic. We further compute a Debye-Waller B-factor by summing self correlations (*x*-*x* and *y*-*y*) and multiplying the sum by 8*π*^2^. For the upper and lower lattices we get 3 × 10^3^ pm^2^ and 3.5 × 10^3^ pm^2^ respectively, which are approximately 6 times lower than the reported value of 2 × 10^4^ pm^2^ obtained from powder X-ray diffraction^38^. Such a discrepancy cannot simply be explained by a lack of *z*-vibrations in our reconstruction, as the *z*-resolution of 2 nm in this ptychographic reconstruction is clearly not sufficient to detect fluctuations along this axis. Although for a 2D material, such as hBN, where the layers are bound only by the Van der Waals forces to one another, the *z*-vibrations might significantly increase the isotropic squared displacements, two additional limiting factors are responsible for reducing the amplitude of reconstructed correlations and the remaining broadening of the images of projected atom positions within the layers: (1) The *z*-distance between atomic layers in hBN is 0.33 nm, which means that per 0.48 nm thin potential slice there are on average 1.4 layers of atoms, the positions of which are averaged over, and (2) the limited number of only 10 object states may not be sufficient to represent all of the TDS and Debye-Waller factor by an incoherent sum of diffraction patterns from statically displaced atom configurations.

Still, despite an underestimated correlation strength, their directions provide direct access to the dynamical matrix of the crystal. Following the procedure described in ref. ^18^, we compute Fourier-transformed displacements by summing over all *L* available unit cells *l*:

5 $${\widetilde{u}}_{\kappa \alpha }(q)=\frac{1}{\sqrt{L}}{\sum }_{l=1}^{L}{u}_{l\kappa \alpha }\times {e}^{-iq{r}_{l}}.$$

By assuming boron’s and nitrogen’s harmonically averaged atomic mass of 12.2 a.m.u. and a temperature of 300 K we obtain the dynamical matrix^18,31^ via:

6 $${D}_{\kappa \alpha {\kappa }^{{\prime} }\beta }(q)=\frac{{k}_{B}T}{m}{\left[\langle {\widetilde{u}}_{\kappa \alpha }(q){\widetilde{u}}_{{\kappa }^{{\prime} }\beta }^{*}(q)\rangle \right]}_{\kappa \alpha {\kappa }^{{\prime} }\beta }^{-1},$$

where *κ*, ${\kappa }^{{\prime} }$ indicate one of two basis atoms and *α*, *β* indicate the displacement directions (*x* and *y*). Computing the square root of eigenvalues of this matrix directly results in the phonon-dispersion curves. Although the 59 unit cells available in our scanned field of view are not sufficient to examine the curves in detail, we compute an average frequency along the *Γ* − *K* − *M* − *Γ* path. We obtain 10.7 THz, 14.1 THz, 17.7 THz and 24.0 THz for transverse acoustic, longitudinal acoustic, transverse optical, longitudinal optical branches in the upper lattice. For the bottom lattice we get 10.8 THz, 13.0 THz, 18.2 THz, and 27.0 THz for the same four branches. The reported theoretical values are approximately 1.5 times higher: 14.3 THz, 22.9 THz, 39.5 THz and 39.6 THz^39^. The scaling of energies calculated via CAVIAR is affected by both assumed temperature as well as extracted amplitude of atomic vibrations. Although the electron beam heats the sample during data collection, this effect is negligible, as the temperature raises only by up to a few tens of Kelvin, which is not enough to observe a noticeable change in energy. The vibration amplitude is a far more important factor, and our simulations show that CAVIAR can underestimate it. Still, CAVIAR makes it possible to place frequencies of two acoustic and two optical branches in the correct range for a complementary Vibrational STEM-EELS measurements^23,40^. Further, unlike EELS, mixed-object ptychography yields directions of correlated vibrations at the level of a single atom.

## Discussion

The experimental discovery of thermal streaks in electron diffraction patterns^41,42^ is as old as the idea of ptychography^1–4^. The impact of lattice vibrations on electron diffraction was heavily investigated both experimentally and theoretically during the last century (e.g., ref. ^43^) and is still a hot research topic today (e.g., ref. ^44^). It has already been pointed out that the faint structure in the diffuse Kikuchi diffraction intensity between the Bragg spots of electron diffraction patterns makes it possible to distinguish between correlated (consistent with the details of the phonon dispersion curve) and uncorrelated (according to the Einstein model) lattice vibrations^17^. Utilizing the computational power and recent advancements in ptychographic reconstruction algorithms, we have extended electron ptychography beyond static imaging by introducing the CAVIAR reconstruction framework capable of resolving spatial correlations in atomic displacements. By coupling a statistical object model^14^ with lattice Green’s function analysis^18^, we recover correlated atomic vibrations directly from experimental 4D-STEM data initially having no energy resolution. We report on deep sub-Å ngstrom spatial resolution and its ratio to the illumination wavelength of *d*/*λ* = 9.78. Simulations presented above reveal that CAVIAR distinguishes between different kinds of lattice vibrations including correlated, uncorrelated, and absent motion, while experimental validation on a twisted hBN bicrystal demonstrates the ability to extract real-space correlation patterns and infer local phonon dispersion. This approach opens a pathway for imaging lattice dynamics and thermal disorder, offering a complementary technique to vibrational STEM EELS^23,40^.

## Methods

### Experimental conditions

The 4D-STEM dataset of hBN bicrystal was acquired in a Nion HERMES microscope using a single 256 × 256 pixel wide chip of the Dectris ELA direct electron detector. The acquisition time, beam current, accelerating voltage, beam convergence semi-angle and the real-space scan step were set to 2 ms, 19 pA, 60 kV, 40 mrad and 30.4 pm, respectively. Diffraction patterns were recorded with a reciprocal sampling of 0.61 mrad per detector pixel. In total, 7 identical 4D-STEM datasets were acquired one after another for the same region of interest and non rigid registration^45^ was applied to eliminate the sample drift. One of these 7 datasets was used to perform the ptychographic reconstruction presented in this paper. In order to exclude inelastic scattering beyond phonon excitation from contributing to the recorded diffraction intensities, we further employed zero-loss energy-filtering. In order to correct for residual geometric distortion created by the energy filter in the recorded diffraction patterns, we acquired two tilt series with a small convergence semi-angle using the ELA detector (placed after the energy filter) and a detector placed before the filter. Using two tilt datasets we fitted a distortion vector field and applied the respective inverse transformation to the recorded diffraction patterns.

### Ptychographic reconstruction algorithm

All ptychographic reconstructions use code written in-house^46^ which employs the Python library CuPy^47^. All reconstructions were done on a single NVIDIA H100 GPU with 96 GB RAM. The code runs a gradient-based minimization of a loss function which implements a metric^25,48,49^ that is based on the discrepancy between the measured intensities *I*^*m*^ and the ones predicted by the forward model, as defined in the Supplementary Eq. 7 and further denoted as *I*. For this study we selected the Gaussian-likelihood metric^29^:

7 $${{{\mathcal{L}}}}_{Gauss}\left(V(\vec{r})\right)=\sum {\left(\sqrt{{I}^{m}}-\sqrt{I}\right)}^{2},$$

where the summation is over all beam positions and all pixels.

The loss derivative and corresponding update-vectors, are computed via Wirtinger calculus^50^ a limited-memory Broyden-Fletcher-Goldfarb-Shanno (l-BFGS) algorithm^50,51^. For the simulated silicon data no additional regularization was employed and an initial guess for the object was created using a uniform prior. The reconstruction shown in Fig. 2e of the main text was carried out in two stages: (1) An initial reconstruction with a single object mode and 9 probe modes initialized using Hermite polynomials^10^; (2) A final reconstruction using a probe fitted during the first stage with 20 object modes. This scheme allows to apply a constraint on the recovered correlations by absorbing as much collective motion of the beam or sample during the first stage and recover the non-uniform vibrations during the second one. The real-space wavefront of probe modes recovered from the silicon dataset with partial spatial coherence are shown in Supplementary Fig. 6.

The experimental hBN dataset was padded with zeros in reciprocal space up to a shape of 732 × 732 pixels resulting in a pixel size in the ptychographic reconstruction of 10.89 pm. We further applied a 2/3 frequency cutoff to all intermediate waves (i.e., between all slices) to prevent aliasing artifacts^52^. The reconstruction went in four stages: (1) An initial reconstruction was run with a single probe mode, a single object mode and 30 object slices. This allowed us to estimate the aberrations of the beam. F arther, cross-correlating the slices with their next neighbors allowed us to determine a minuscule difference between the zone axis of the crystal and the beam propagation direction. We used this value and adopted a tilted propagator^53^ as defined in Supplementary Eq. 5 without an additional refinement of the angle for all subsequent steps. A position-averaged diffraction pattern demonstrating the amount of misorientation is shown in Supplementary Fig. 8. Multiple probe modes could have been used during this stage. However, for the sake of reconstruction speed, they were introduced in the next stage. (2) Using the angle fitted during stage 1 a single object mode was reconstructed with 10 probe modes and without any regularization ; (3) The five dominant probe modes out of ten total modes recovered during the second stage were selected and 10 object modes were generated by adding a uniform prior (noise) to the object recovered at stage 2; (4) After the object at stage 3 was free from noise we added at the beginning, we applied regularization to the object, in particular, an *l*_1_-regularization^25^ to the absorption potential enforcing the amplitude of the retrieved transmission function to be closer to 1, and also a missing-wedge regularization^10,53^ which dampens high *k*_*z*_ Fourier-components at small *k*_*x*_ and *k*_*y*_. The probe modes recovered after the final stage of a reconstruction from the experimental hBN dataset are shown in Supplementary Fig. 7.

## Supplementary information

Supplementary Information

Transparent Peer Review file

## Data Availability

The simulated and experimental 4D-STEM datasets, together with the corresponding ptychographic reconstructions presented in this study, have been deposited on the Code Ocean platform^54^ under the accession code 10.24433/CO.7393167.v3.

## Code availability

The custom-developed ptychographic reconstruction software and analysis tools used in this study are openly available on the Code Ocean platform^54^. The source code for the ptychographic reconstruction engine is also hosted on GitHub^46^ at https://github.com/Anton-Gladyshev/pypty.
