# Supplementary material for: Electron ptychography reveals correlated lattice vibrations at atomic resolution
Source: Nat Commun. 2026 Jun 10;17:5173. doi: 10.1038/s41467-026-74135-4 (PMC13254316; doi:10.1038/s41467-026-74135-4)
Supplement: Supplementary file 1 — Supplementary Information [file 41467_2026_74135_MOESM1_ESM.pdf]

Supplementary Information for Electron Ptychography Reveals Correlated Lattice Vibrations at Atomic Resolution

Anton Gladyshev, Benedikt Haas, Thomas C. Pekin, Tara M. Boland, Marcel Schloz, Peter Rez and Christoph T. Koch
May 26, 2026

Theory

Strictly speaking, an iterative ptychographic reconstruction algorithm fits a forward model that, for a given beamposition (two coordinates $\rho_{p,x}$ and $\rho_{p,y}$) maps an illumination wavefront to a measured diffraction pattern given in terms of spatial frequencies k_x and k_y. This model includes a transmission function of the investigated sample and can be formulated in various complexity levels. Recovering the initially unknown transmission function is the main goal of any ptychographic reconstruction, as its amplitude characterizes absorption while its phase is directly proportional to the specimen's electrostatic potential. During the reconstruction one can additionally refine the probe [1, 2, 3], scan positions [2, 3, 4] and/or a mis-tilt angle between the optical axis of the microscope and the zone axis of the studied crystal [5].

When an electron beam passes through a sufficiently thin sample, it may be assumed to undergo at most one scattering event. The three dimensional structure of an object can then be reduced to just two lateral dimensions by integrating over the beam propagation direction. For a beam position ρ_p, the exit wave $\psi^{(exit)}(\rho_p, \rho)$ becomes a real space product of a two-dimensional wave function of the incident beam $\psi^{(in)}(\rho - \rho_p)$ with a two-dimensional complex transmission function of the specimen $O(\rho)$. The corresponding diffraction pattern (far-field intensity) can be calculated as the squared modulus of the Fourier-transformed exit wave:

$$I(\rho_p, k) = \left|\mathcal{F}\left\{\psi^{(exit)}(\rho_p, \rho)\right\}\right|^2 = \left|\mathcal{F}\left\{\psi^{(in)}(\rho - \rho_p) \cdot O(\rho)\right\}\right|^2, \quad \text{(S1)}$$

where $\mathcal{F}$ denotes a Fourier transform. Increasing the beam's wavelength and specimen thickness makes the effect of multiple scattering more pronounced. Ref. [6] showed that at some point the thin object approximation starts to fail. In this case the most efficient strategy is to "divide and conquer". Instead of using one two dimensional transmission function, one can split the propagation into multiple intervals and define a set of 2D transmission functions responsible for each particular sufficiently thin region. We can write

$$\psi_j^{(exit)}(\rho_p, \rho) = \psi_j^{(in)}(\rho - \rho_p) \cdot O_j(\rho), \quad \text{(S2)}$$

where j indicates a particular interval, i.e. slice. The propagation between neighboring slices j and $j+1$ over the interval d is computed using the convolution with the Fresnel propagator:

$$\psi_{j+1}^{(in)}(\rho) = \mathcal{F}^{-1}\left\{\mathcal{F}\left\{\psi_j^{(exit)}(\rho)\right\} \cdot \mathcal{P}_{Fr}(k)\right\} \quad \text{(S3)}$$

$$\mathcal{P}_{Fr}(k) = \exp\left[-i\pi\lambda d|k|^2\right], \quad \text{(S4)}$$

where λ is the wavelength of the electron beam and equation S4 defines the Fresnel propagator in reciprocal space. Typically one chooses the distance between the slices to be approximately 1 nm [6, 7]. In this manner the

incident illuminating wavefront $\psi^{(in)}_{j=0}(\rho-\rho_p)$ is propagated through N slices, and the exit-wave $\psi^{(exit)}_{j=N}(\rho)$ is then used to calculate a diffraction pattern as described in equation S1. Often the beam propagation direction slightly deviates from the zone axis of the crystal. Then, the Fresnel propagator can be modified [8, 5] to compensate for misalignment angles up to a few degrees. For two misalignment angles α_x and α_y along x and y axes, respectively, the tilted Fresnel propagator is defined as follows:

$$\mathcal{P}_{Fr}\left(k,\alpha_x,\alpha_y\right)=\exp\left[-i\pi\left(\lambda d|k|^2+2k_x\alpha_x+2k_y\alpha_y\right)\right]. \tag{S5}$$

In a real experimental situation, it is not always appropriate to neglect the partial spatial coherence of the electron source and vibrations of the atoms. To account for partial spatial coherence, Thibault and Menzel [9] proposed to replace the pure probe state $\psi^{(in)}_{j=0}(\rho)$ with a statistical mixture of multiple probe states $\psi^{(in)}_{j=0,m}(\rho)$, where the first index $j=0$ remained from the multi-slice formalism and the second index m accounts for multiple modes. The total predicted diffraction pattern is calculated as an incoherent sum of the intensities corresponding to the individual probe modes. Let $I^{(1)}(\psi^{in}_{j=0}(\rho-\rho_p), O(\rho))$ denote the sequence of operations required to obtain a diffraction pattern from a single probe mode. In mixed-probe formalism, the intensity is modeled as

$$I_{total}=\frac{1}{N_{probe\ modes}}\sum_{m=0}^{N_{probe\ modes}} I^{(1)}(\psi^{in}_{j=0,m}(\rho-\rho_p), O(\rho)). \tag{S6}$$

The mixed-object formalism [9] is a natural extension of the mixed-probe formalism that accounts for a non-stationary object's transmission function. Due to the lattice vibrations and the corresponding displacements of the atoms, two electrons hitting the specimen at exactly the same spatial position but at two different points in time interact with slightly different electrostatic potentials. To account for this effect, i.e. thermal diffuse scattering (TDS) [10], one can use multiple transmission functions and model a diffraction pattern as an incoherent average of the intensities corresponding to the individual pure transmission functions.

$$I_{total}=\frac{1}{N_{object\ modes}\cdot N_{probe\ modes}}\sum_{n=0}^{N_{object\ modes}}\sum_{m=0}^{N_{probe\ modes}} I^{(1)}(\psi^{in}_{j=0,m}(\rho-\rho_p), O_n(\rho)). \tag{S7}$$

Thus, in the most complex scenario one has to deal with a three dimensional illumination wavefront (2 lateral dimensions plus one dimension for multiple modes), a four dimensional object (2 lateral dimensions and two dimensions one each for multiple slices and multiple modes) and perform $N_{object\ modes}\times N_{probe\ modes}$ forward multi-slice propagations to model one diffraction pattern.

Undersampling along the Beam Propagation Direction

We would like to highlight an importance of accounting for multiple scattering when performing mixed-object ptychography. Reconstructions presented in the main text were done using a slice thickness of 2 Å, but the corresponding simulations used a slice thickness of 0.5 Å. The reason for such fine sampling in the reconstruction lies in the fact, that the effect of under-sampling along the beam propagation direction may be much more noticeable than the features produced by a particular type of atomic motion. We performed additional 4D-STEM simulations using the same 30 MD snapshots of the silicon grain boundary but with an increased slice thickness. In Fig. 1a we show the integrated position-averaged Gaussian-log likelihood (in arbitrary units) between one 4D-STEM dataset simulated for correlated atomic displacements and a slice thickness of 0.5 Å and datasets simulated also for correlated atomic displacements but with larger slice thicknesses. The gray dashed line shows the Gaussian log-likelihood between 4D-STEM simulations from correlated and uncorrelated atomic displacements, both simulated using a slice thickness of 0.5 Å(see also Fig. 1b). It clearly follows from the plot that the difference between various kinds of interatomic vibrations is far weaker than the difference caused by undersampling along the beam propagation direction. In Fig. 1b – d we show differences between position-averaged diffraction patterns (PACBED) of simulations from models with correlated and uncorrelated displacements, correlated and undersampled-correlated displacements, and correlated displacements and no displacements at all, i.e. a perfectly coherent datasets, respectively.

Benchmarking Temporal Resolution and Recovery of Interatomic Vibrations

The CAVIAR reconstructions from a simulated 4D-STEM dataset of a silicon grain boundary, shown in Fig. 2 of the main text, demonstrate that while the directions of atomic vibrations can be retrieved with relatively little

information loss, the strength of the interatomic correlations is significantly underestimated. To further quantify this effect, we performed additional calculations using the raw molecular dynamics snapshots that were used to generate the 4D-STEM datasets. The initial frames were sampled with a 1 fs step [11]. Gaussian blurring was sequentially applied along the time axis with various standard deviations, effectively degrading the time resolution, and the resulting correlations were compared with unblurred data. The curves showing the response of the length similarity metric (LSM) and the cosine similarity metric (CSM), defined in Eqs. (3) and (4) of the main text, respectively, to time resolution are presented in Fig. 3. As the time resolution decreases, i.e., with increasing standard deviation, the LSM values drop exponentially, whereas the CSM curve resembles an arccotangent function, decreasing much more slowly. The plots show that a standard deviation of 0.67 fs yields an LSM value of 0.56 and a CSM value of 0.95, closely matching the benchmarks achieved by the CAVIAR reconstruction presented in Fig. 2e of the main text. While the origin of the time resolution still has to be investigated, it might be linked to the ratio between the detector pixel size, i.e. Fourier space sampling, and the size of features in the diffraction pattern that are unique for a particular set of interatomic correlations [12].

It has been shown that, for simulations of electron diffraction patterns, thermal diffuse scattering effects can be accounted for by using a few tens of atomic configurations, i.e. object states [13]. To benchmark the response of the CAVIAR reconstruction to the number of object states used, we performed an additional ptychographic reconstruction from the experimental 4D-STEM dataset of a twisted hBN bicrystal using 20 object states and 5 probe states. The initial guess for the object was created by stacking two copies of the result obtained from the 10-state reconstruction and adding a small amount of uniform prior to introduce initial variation across the states. Upon convergence, the interatomic correlations as well as the phonon dispersion curves were extracted via CAVIAR. The results of the 20-state reconstruction are presented in Fig. 5, using a similar style to that used for the 10-state reconstruction in Fig. 4. While the entries of the Green's tensor appear slightly less noisy in the 20-state reconstruction, the corresponding phonon dispersion curves do not show significant changes. Furthermore, the average energies of four branches remain approximately 1.5 times lower than reported theoretical values [14] and show no improvement. Taken together with the time-resolution dependence of the LSM metric, these results suggest that time blurring, rather than the number of object states, is the dominant factor limiting the reconstruction quality. Moreover, in case of the experimental data the extracted interatomic correlations may be further degraded by Poisson noise present in the diffraction patterns.

Remarks on a Full Three Dimensional Ptychographic Reconstruction

A truly full 3D reconstruction from data recorded for just a single sample orientation is not possible, as the lowest spatial frequencies along the beam direction are lost due to the finite convergence angle of the illumination [15, 16]. However, although very challenging experimentally, the determination of 3D atomic positions from a single-tilt 4D-STEM dataset may still be theoretically possible. Unlike in projection imaging, the minimal realistic interatomic distance in three dimensions is around 2 Å. Achieving such depth resolution for median in-plane spatial frequencies between $1/2$ Å^{-1} and 1 Å^{-1}, corresponding to in-plane resolutions of 2 Å and 1 Å, respectively, requires convergence semi-angles in the range between $\arctan(0.5/2)$ and $\arctan(0.5/0.1)$, approximately 245 and 464 mrad. While angles as large as 0.464 rad are likely unrealistic experimentally, the lower bound of 245 mrad may become feasible in the future. While aberration correctors are currently able to reduce aberrations up to approx. 50 mrad, they increase aberrations beyond that. It may thus be preferential to perform such experiments in an uncorrected TEM. Sub-A ptychographic reconstructions using such instrument has recently been demonstrated [17]. From our perspective, a far more critical limitation is the number of pixels in hybrid-pixel direct electron detectors, as well as the acceptance angles of electromagnetic lenses and apertures. While there are no fundamental physical constraints preventing such a configuration of an electron microscope, the required development would likely be technically challenging, and we are not aware of any ongoing engineering efforts aimed at extending this angular range.

Extended data figures

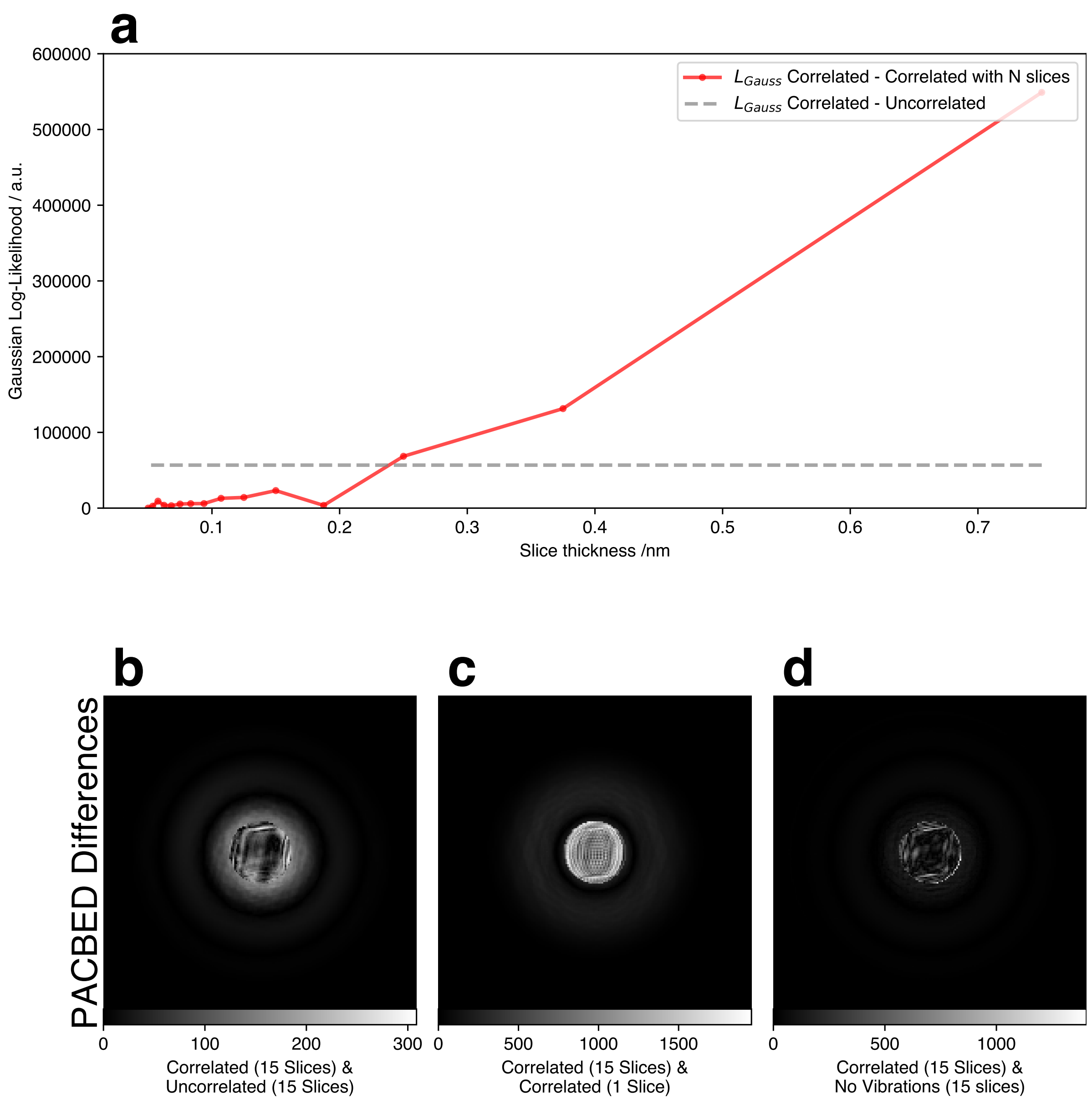

Supplementary Figure 1. Effect of depth undersampling, i.e. of not sufficient slices along the beam propagation direction. Panel **a** shows the Gaussian log-likelihood between a fully sampled correlated 4D-STEM dataset (0.5 Å thick slices) of a symmetric $\Sigma 9$ grain boundary in silicon and multiple undersampled correlated datasets with increasing slice thickness (red), as well as between the correlated and uncorrelated datasets (gray dashed). Panels **b–d** show PACBED difference maps comparing: **b** correlated vs uncorrelated displacements (15 slices), **c** finely sampled (15 slices) vs undersampled (1 slice) displacements, and **d** correlated displacements vs. no displacements at all. Artifacts from undersampling exceed those caused by correlations in the interatomic displacements. Thus, to recover vibrations one should first account for the effect of multiple scattering, even with a 0.7 nm thin sample.

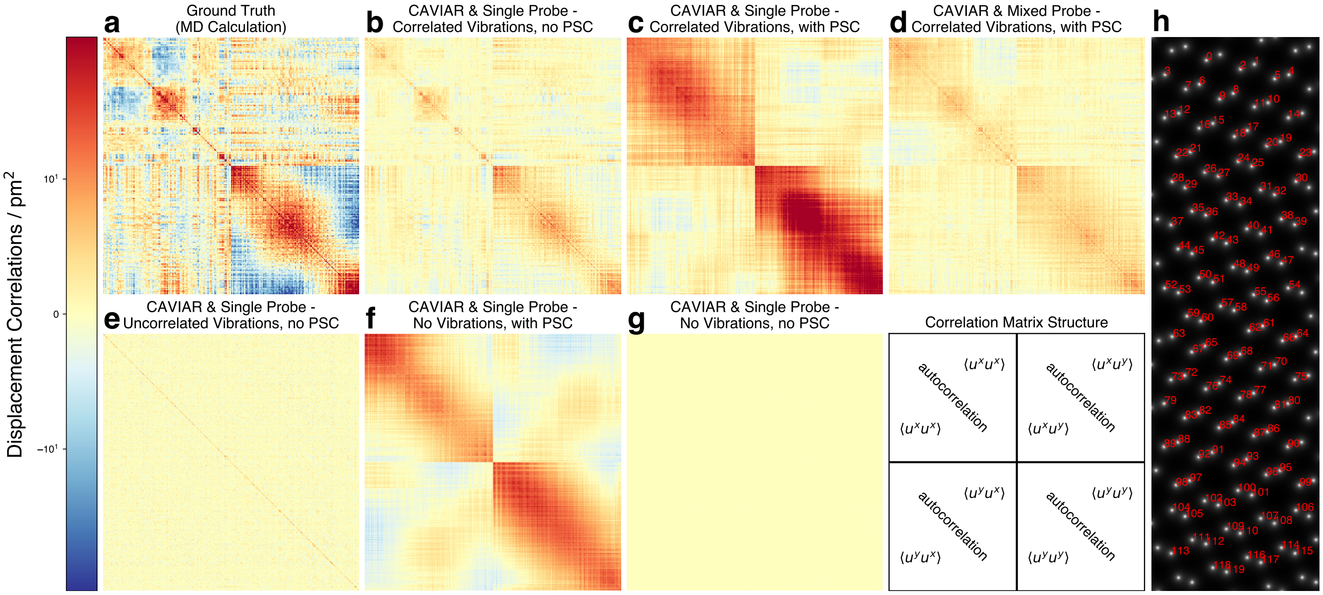

Supplementary Figure 2. Full Green's tensors obtained via CAVIAR from various 4D-STEM datasets of a symmetric Σ9 grain boundary in silicon. a, The ground truth correlation matrix derived from molecular dynamics simulations with clear off-diagonal structure indicating spatially correlated atomic displacements. **b,** The result of a CAVIAR reconstruction from the dataset with correlated vibrations and no partial spatial coherence (PSC), preserving the main features of the ground truth but with slightly lower magnitude. **c,** CAVIAR reconstruction using a single probe mode from a dataset simulated with correlated atomic displacements with additional partial spatial coherence, producing a structure similar to Panel **a** but offset by a uniform background. **d,** CAVIAR reconstruction from the same data as in **c**, but using 9 probe modes. The mixed probe approach efficiently mitigates the mentioned offset in correlations and produces correlations very similar to the ones shown in **a** and **b**. **e,** The reconstruction from a dataset simulated for displacements based on the Einstein model without additional PSC, off-diagonal elements of the Green's tensor are close to zero indicating an absence of correlations between different degrees of freedom. **f,** A reconstruction from a dataset without any atomic vibrations but with partial spatial coherence (the dataset was not presented in the main text of the paper). The observed correlations stem from the incoherent illumination rather than atomic motion illustrating a positive offset mentioned the main text of the paper. **g,** The result for a perfectly static sample without PSC, where the correlation matrix exhibits near-zero values throughout, reflecting the absence of motion. In panel **h** we show indexing of atoms on top of a ground-truth phase. The structure of the matrices is schematically shown in the lower right corner. All CAVIAR reconstructions **b – g** except **d** used a single probe mode, in **d**, 9 incoherent probe modes were used.

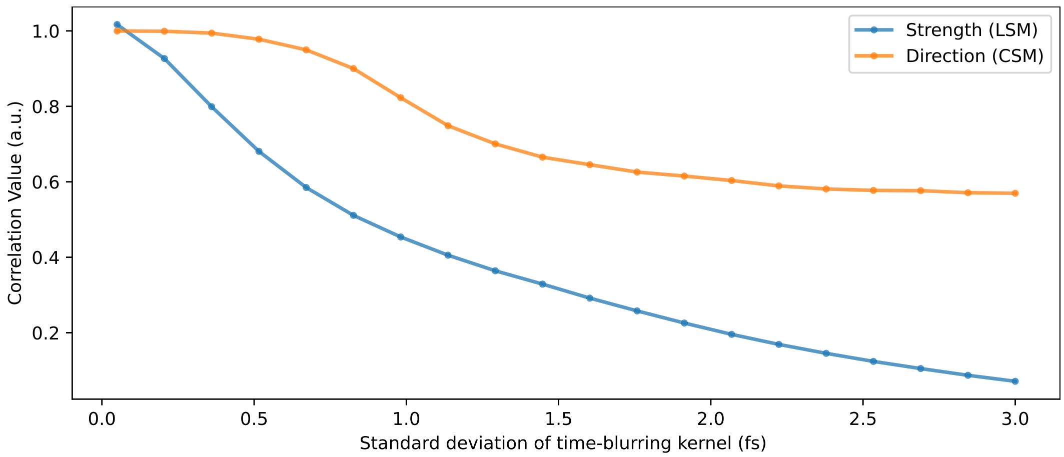

Supplementary Figure 3. Dependence of benchmarking metrics for strength (LSM) and direction (CSM) of reconstructed atomic vibrations on time resolution. The orange and blue curves illustrate the effect of applying Gaussian blurring along the time axis to molecular dynamics snapshots of a symmetric $\Sigma 9$ grain boundary in silicon, showing how temporal smoothing alters the extracted strength and direction of atomic vibrations compared to unblurred data with 1 fs time sampling. The LSM and CSM metrics are defined in equations (3) and (4) of the main text, respectively.

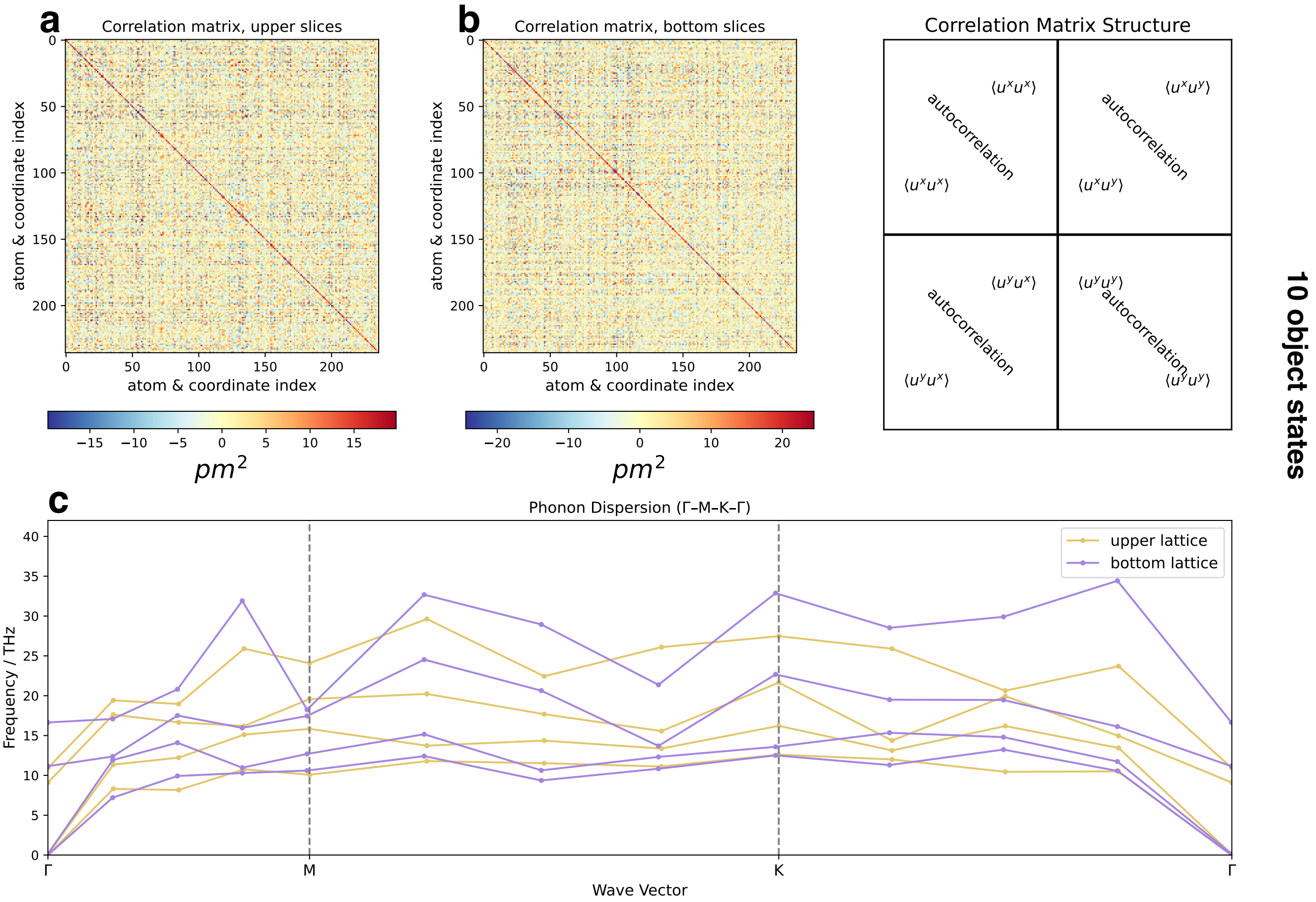

Supplementary Figure 4. Interatomic correlations (Green's tensors) recovered from a 10-state mixed-object ptychographic reconstruction of an experimental 4D-STEM dataset of the approximately 15 nm thick hBN bicrystal. Images **a** and **b** show average correlation matrices extracted from the z-ranges $z = 1.9$ nm $\ldots 5.7$ (top lattice) and $z = 8.6$ nm $\ldots 12.5$ nm (bottom lattice), respectively. The indices of the matrices run over all atoms available in the field of view and two spatial coordinates (x, y), the structure of the matrices is schematically depicted on the right side. **c**, Phonon dispersion curves extracted from the reconstructed atomic displacement correlations using the lattice Green's tensor method. Yellow curves show the dispersion from the upper hBN crystal, and purple curves the one from the lower crystal. Both are computed along the Γ-M–K-Γ direction of the Brillouin zone. The curves reveal acoustic and optical branches, although their absolute frequencies are reduced due to limited sampling of object states, limited spatial resolution and the lack of z-displacement sensitivity in the 2D projection. In the upper lattice the average frequencies along the $\Gamma - K - M - \Gamma$ path are 10.7 THz, 14.1 THz, 17.7 THz and 24.0 THz for transverse acoustic, longitudinal acoustic, transverse optical, longitudinal optical branches in the upper lattice. For the bottom lattice we obtain 10.8 THz, 13.0 THz, 18.2 THz, and 27.0 THz for the same four branches.

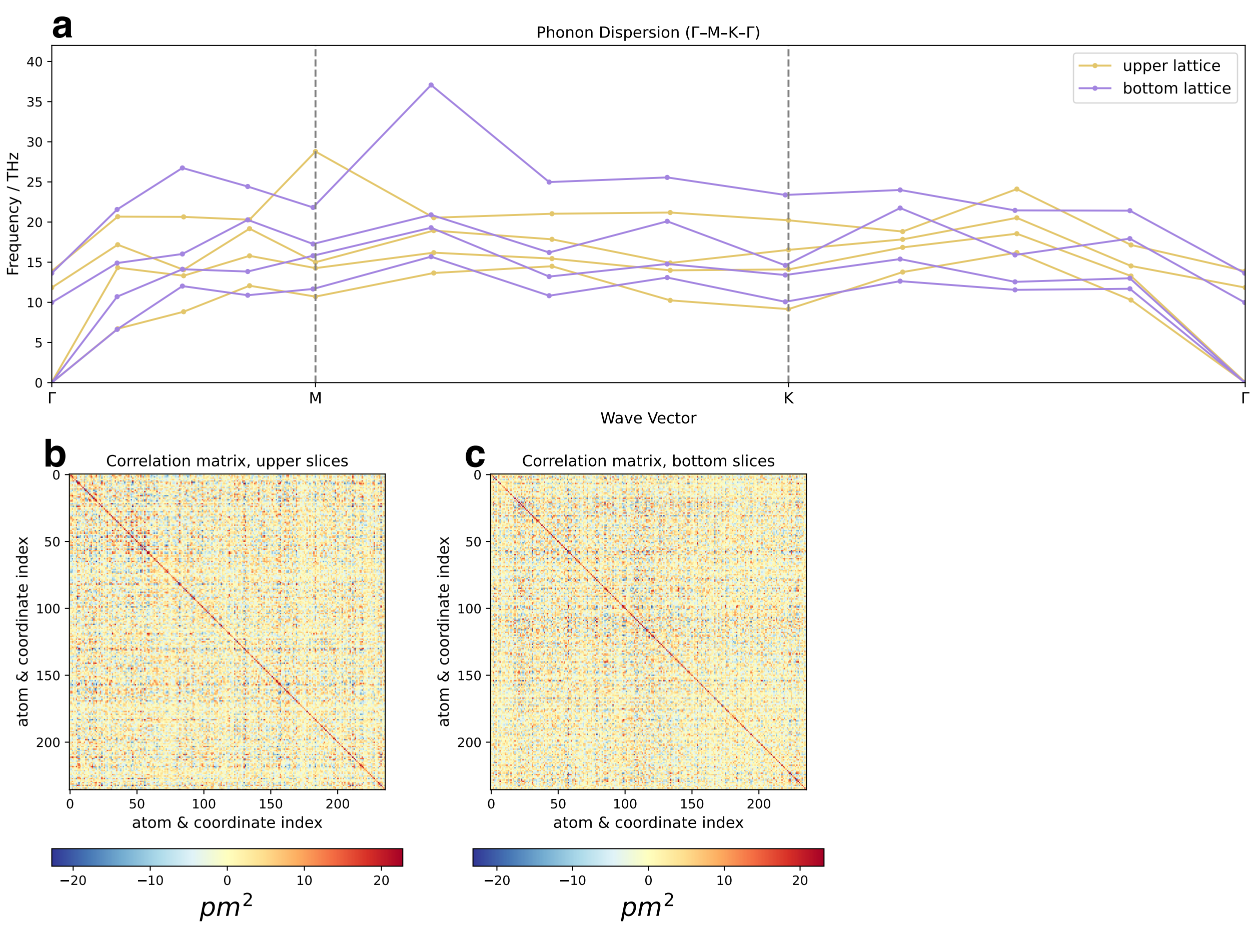

20 object states

Supplementary Figure 5. Interatomic correlations (Green's tensors) recovered from experimental 20-state mixed-object ptychographic reconstruction of an approximately 15 nm thick bulk hBN crystal. a, Phonon dispersion curves extracted from the reconstructed atomic displacement correlations using the lattice Green's tensor method from the experimental bulk hBN dataset. Yellow curves show the dispersion from the upper hBN lattice layer, and purple curves the one from the lower layer. Both are computed along the Γ-M–K-Γ direction of the Brillouin zone. In the upper lattice the average frequencies along the $\Gamma - K - M - \Gamma$ path are 11.5 THz, 15.1 THz, 17.0 THz and 21.2 THz for transverse acoustic, longitudinal acoustic, transverse optical, longitudinal optical branches in the upper lattice. For the bottom lattice we get 11.5 THz, 14.2 THz, 17.8 THz, and 24.8 THz for the same four branches. Panels **b** and **c** show average correlation matrices extracted from the z-ranges $z = 1.9$ nm $\ldots 5.7$ (top lattice) and $z = 8.6$ nm $\ldots 12.5$ nm (bottom lattice), respectively.

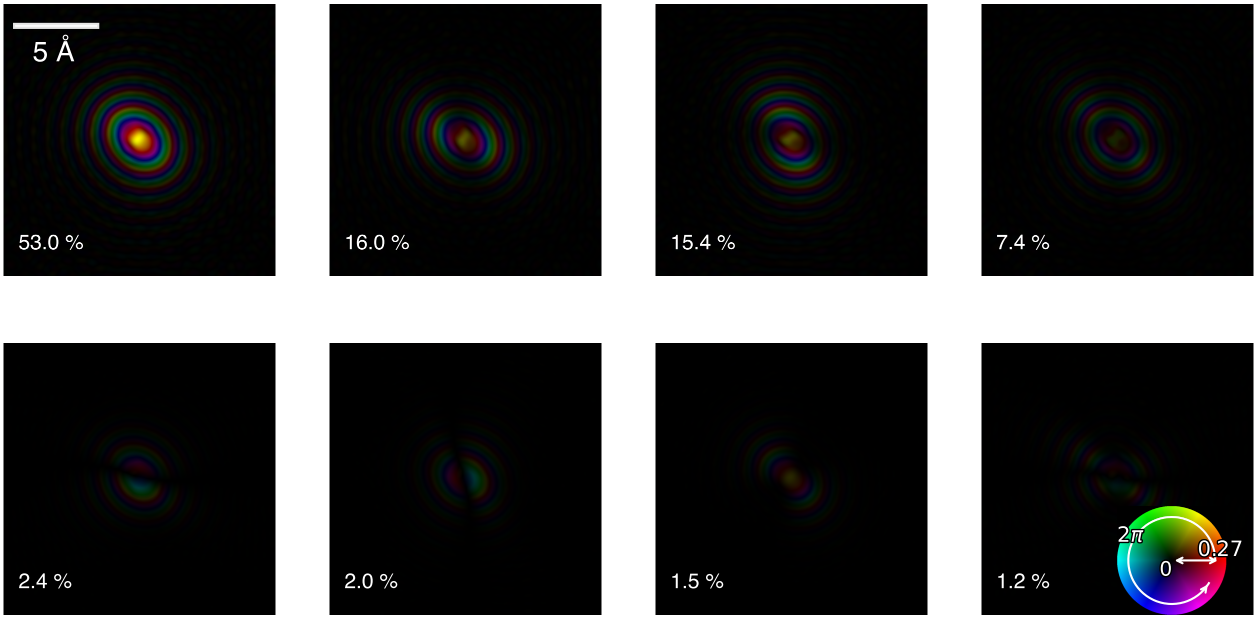

Supplementary Figure 6. Real-space wavefronts of probe modes reconstructed from the simulated 4D-STEM dataset of the symmetric $\Sigma 9$ grain boundary model in silicon, including the effect of partial spatial coherence. Eight dominant out of the nine modes are shown using a complex colormap, where color represents the phase and brightness denotes the amplitude. The percentage of the total intensity captured by each mode is reported in the lower left corner of each image.

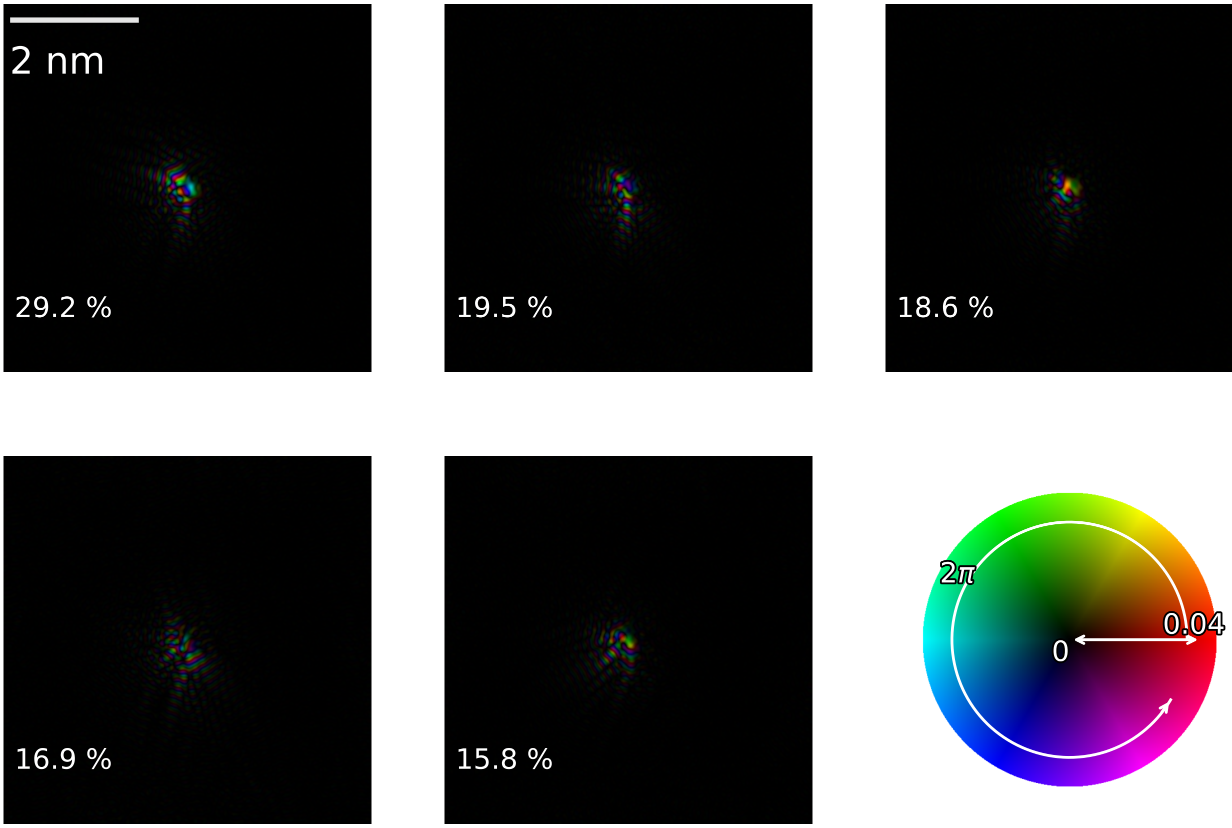

Supplementary Figure 7. Real-space wavefronts of probe modes reconstructed from the experimental 4D-STEM dataset of the twisted hBN bicrystal. All five reconstructed modes are shown using a complex colormap, where color represents the phase and brightness denotes the amplitude. The percentage of the total intensity captured by each mode is reported in the lower left corner of each image.

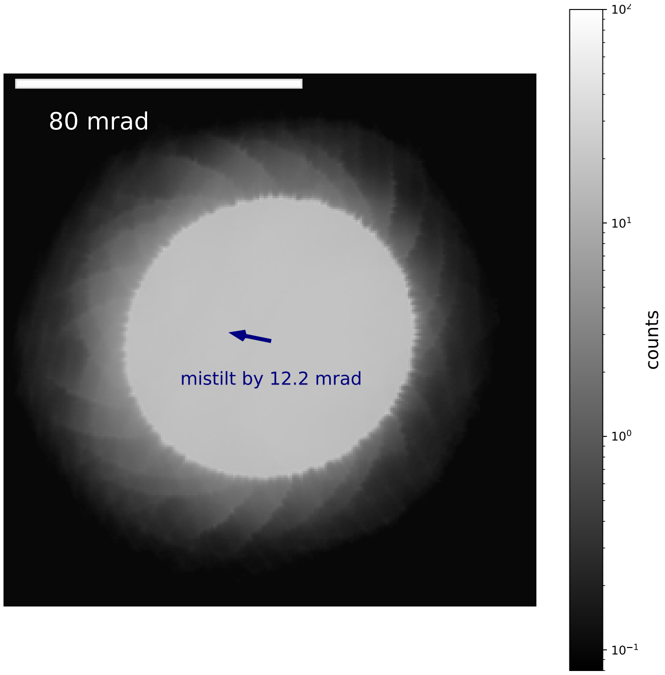

Supplementary Figure 8. Position-averaged convergent-beam electron diffraction pattern (PACBED) obtained by real-space averaging of the 4D-STEM dataset of the twisted hBN bicrystal. The mistilt angle of 12.2 mrad, manifested by the shift of the scattered discs and calculated from a ptychographic reconstruction, is indicated by the blue arrow.

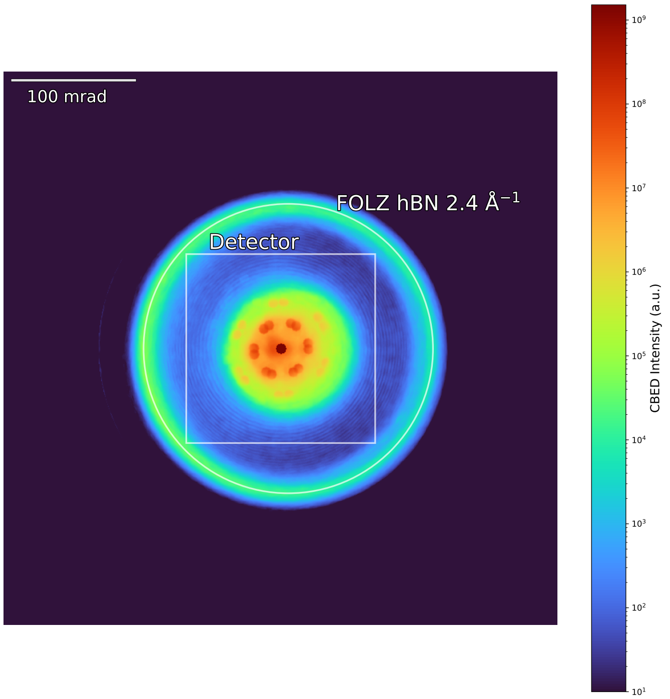

Supplementary Figure 9. Position averaged convergent-beam electron diffraction (PACBED) pattern simulated by multislice propagation of a convergent electron probe through the potential slices that were ptychographically recovered from the experimental 4D-STEM data set of the twisted hBN bicrystal. A beam energy of 60 kV and a convergence semi-angle of 4 mrad were assumed. The first-order Laue zone (FOLZ) ring, visible at 2.4 Å^{-1}, indicates that for high in-plane spatial frequencies the achieved depth resolution is better than 6.7 Å, corresponding to the out-of-plane unit cell parameter c of hBN. The square indicates the range of spatial frequencies recorded by the detector during the experiment.

References

[1] Maiden, A. & Rodenburg, J. An improved ptychographical phase retrieval algorithm for diffractive imaging. *Ultramicroscopy* **109**, 1256–1262 (2009).

[2] Schloz, M. *et al.* Overcoming information reduced data and experimentally uncertain parameters in ptychography with regularized optimization. *Optics express* **28**, 28306–28323 (2020).

[3] Du, M. *et al.* Adorym: A multi-platform generic x-ray image reconstruction framework based on automatic differentiation. *Optics express* **29**, 10000–10035 (2021).

[4] Maiden, A., Humphry, M., Sarahan, M., Kraus, B. & Rodenburg, J. An annealing algorithm to correct positioning errors in ptychography. *Ultramicroscopy* **120**, 64–72 (2012).

[5] Sha, H., Cui, J. & Yu, R. Deep sub-angstrom resolution imaging by electron ptychography with misorientation correction. *Science Advances* **8**, eabn2275 (2022).

[6] Chen, Z. *et al.* Electron ptychography achieves atomic-resolution limits set by lattice vibrations. *Science* **372**, 826–831 (2021).

[7] Brown, H. G. *et al.* A three-dimensional reconstruction algorithm for scanning transmission electron microscopy data from a single sample orientation. *Microscopy and Microanalysis* **28**, 1–9 (2022).

[8] Kirkland, E. *Advanced Computing in Electron Microscopy* (Springer Nature Switzerland AG, 2020).

[9] Thibault, P. & Menzel, A. Reconstructing state mixtures from diffraction measurements. *Nature* **494**, 68–71 (2013).

[10] Loane, R. F., Xu, P. & Silcox, J. Thermal vibrations in convergent-beam electron diffraction. *Acta Crystallographica Section A* **47**, 267–278 (1991).

[11] Rez, P., Boland, T. M., Elsässer, C. & Singh, A. K. Localized phonon densities of states at grain boundaries in silicon. *Microscopy and Microanalysis* **28**, 1–8 (2022).

[12] Muller, D. A., Edwards, B., Kirkland, E. J. & Silcox, J. Simulation of thermal diffuse scattering including a detailed phonon dispersion curve. *Ultramicroscopy* **86**, 371–380 (2001).

[13] Koch, C. T. *Determination of core structure periodicity and point defect density along dislocations*. Ph.D. thesis, Arizona State University (2002).

[14] Mann, S. & Jindal, V. K. Thermal expansion in 2d honeycomb structures: Role of transverse phonon modes (2016). 1606.07656.

[15] Dong, Z. *et al.* Sub-nanometer depth resolution and single dopant visualization achieved by tilt-coupled multislice electron ptychography. *Nature Communications* **16**, 1219 (2025).

[16] Van den Broek, W. & Koch, C. T. General framework for quantitative three-dimensional reconstruction from arbitrary detection geometries in tem. *Phys. Rev. B* **87**, 184108 (2013).

[17] Nguyen, K. X. *et al.* Achieving sub-0.5-angstrom–resolution ptychography in an uncorrected electron microscope. *Science* **383**, 865–870 (2024). URL https://doi.org/10.1126/science.adl2029.
