# Supplementary material for: Electron ptychography reveals correlated lattice vibrations at atomic resolution
Source: Nat Commun. 2026 Jun 10;17:5173. doi: 10.1038/s41467-026-74135-4 (PMC13254316; doi:10.1038/s41467-026-74135-4)
Supplement: Supplementary file 2 — Transparent Peer Review file [file 41467_2026_74135_MOESM2_ESM.pdf]

nature portfolio

Peer Review File

Electron Ptychography Reveals Correlated Lattice Vibrations at Atomic Resolution

Corresponding Author: Mr Anton Gladyshev

This file contains all reviewer reports in order by version, followed by all author rebuttals in order by version.

Version 0:

Reviewer comments:

Reviewer #1

(Remarks to the Author)
Electron ptychography has delivered record-breaking spatial resolution in numerous materials systems and under challenging experimental conditions (low dose, low-voltage, without Cs-corrector, thick specimen, etc.). Recently, the application of ptychography to reveal lattice-vibration-related features has been explored by several groups, which uncovered unique phenomena inaccessible by other imaging techniques, unleashing a new development thread of computational imaging. In this manuscript, the authors demonstrated that ptychographic reconstructions with multiple states of the object can deliver information about the correlated lattice vibrations. To my best knowledge, it is the first report of ptychographic reconstruction with mixed object states at atomic resolution. The manuscript is well organized and can be published with the following issues addressed.

1. The authors are encouraged to make the code, key raw datasets, and scripts for analysis public.

2. The authors should cite previous work studying the lattice-vibration-related phenomena using ptychography. For example, the recent one by Huang's group (Atom-by-atom imaging of moiré phasons with electron ptychography).

3. The authors used mix-state probes in the reconstruction. So please provide all the probes in the supplementary file. In addition, are the probes updated in the second stage of the reconstruction of Fig. 2e? Please make it clear how the probes are fitted and explain why fitted probes are necessary in the second stage, while not in the first stage.

4. "Further, cross-correlating the slices with their next neighbors allowed us to determine a minuscule difference between the zone axis of the crystal and the beam propagation direction. We used this value and adopted a tilted propagator [6] as defined in S5 without an additional refinement of the angle for all subsequent steps."
First, the authors should provide PACBED showing the degree of off-zone and quantify the deviation. Second, the authors should evaluate how the deviation from the zone axis affects the results.

5. "Moreover, using only the masses of the atomic species and the temperature as input, we obtain average frequencies of 10.8±0.1, 13.6±0.6, 18.0±0.2, 25.5±1.5 THz for the longitudinal and transversal acoustic and optic phonons, respectively, in agreement with inelastic neutron scattering, albeit from just a few nm3 volume."
As the beam irradiation can heat the sample, how does the temperature affect the results?

6. "Fig. 3c and clearly indicates an information transfer within the full convergence angle of the beam (80 mrad) [14, 21]."
In principle, the resolution should be somewhat improved as the sample incoherence is addressed. Why is the resolution still limited to 2 alpha?

7. "The reconstructed transmission function included 10 object states and 30 slices with a spacing of 0.48 nm."
As there is no ground truth for experimental datasets, the authors should test the number of object states.

(Remarks on code availability)

Reviewer #2

(Remarks to the Author)
The paper by Gladyshev et al reports on a further new development in the filed of electron ptychography in which the authors extend the previously reported 3D multislice approach to a 4D mixed object multislice ptychography which provide information on lattice vibrations. As such the paper should certainly be published in nature Communications.

I have some suggestions / questions that the authors might wish to address but these are in the spirit of constructive comments and none of them should preclude publication.

A. The comparison with vibrational EELS is, in my view the most powerful point in the paper but it is somewhat buried late in the introduction. Maybe a mention in the abstract would be helpful. It might also be useful to have an indication of the momentum / spatial limits of the new method and a comparison of these to the limits in Vibrational EELS- an initial crude calculation suggests that access to the phonon spectra should be possible experimentally at close to atomic resolution in x-y and this is highly significant.
B. In Figure 2 (at low resolution) it seems that CAVIAR with correlated vibrations but without FSC gives similar results for the self correlations but that the advantage of including FSC comes through in the correlation with the reference atom and in better fidelity of the angles recovered. Moreover in both cases as the authors note the magnitude of the correlations in all cases is lower that that of the ground truth. The authors suggest that this latter effect is due to a resolution limit in the reconstruction. It might be helpful to compare the reconstructions with a de-resolved (blurred) version of the ground truth. I also missed the temperature of the MD simulations and possible a slight adjustment to this would improve the match in the magnitudes. This would also give an indication of the likely precision recast as an error in the temperature.
C. On p6 (in my copy) it is stated that 10 object states may not be sufficient and it wold help to have an approximate indication of the number required to accurately model the TDS and Debye Waller factor.

Finally the authors correctly point out on p4 that the current depth resolution in STEM is insufficient to recover a full 3D reconstruction. It would be useful to have firstly an indication of the resolution limit required and the electron optical correction needed to achieve this. I suspect that it may not be achievable with current correctors for hard materials that require high depth resolution but there may be interesting applications to soft materials at lower resolution.

Finally to reiterate the above are intended as helpful suggestions in part to satisfy my interest and are not intended to prevent publication of this excellent work

(Remarks on code availability)
The code provided is sufficient to allow others to reproduce that data reported.

Version 1:

Reviewer comments:

Reviewer #1

(Remarks to the Author)
The revised manuscript has fully addressed the questions raised during the first round of review. I recommend it to be accepted for publication. A very nice piece of work.

(Remarks on code availability)

Reviewer #2

(Remarks to the Author)
The author have comprehensively and helpfully address al of the comments raised in my first review. I therefore recommend publication of the paper in its revised form

(Remarks on code availability)

Open Access This Peer Review File is licensed under a Creative Commons Attribution 4.0 International License, which permits use, sharing, adaptation, distribution and reproduction in any medium or format, as long as you give appropriate credit to the original author(s) and the source, provide a link to the Creative Commons license, and indicate if changes were made.
In cases where reviewers are anonymous, credit should be given to 'Anonymous Referee' and the source.
The images or other third party material in this Peer Review File are included in the article’s Creative Commons license, unless indicated otherwise in a credit line to the material. If material is not included in the article’s Creative Commons license and your intended use is not permitted by statutory regulation or exceeds the permitted use, you will need to obtain permission directly from the copyright holder.
To view a copy of this license, visit https://creativecommons.org/licenses/by/4.0/

RESPONSE TO REVIEWERS COMMENTS

We sincerely thank the editor, Reviewer #1 and Reviewer #2 for their careful evaluation of our manuscript and for providing insightful and constructive feedback. Their comments have helped us improve the clarity, rigor, and presentation of the work.

We have copied the referees ’comments from the e-mail and placed them in black text in the pages that follow.

> Our responses are indented and coloured blue.

Parts which have been copied from the main text are not indented but are marked with a bar on the left side as shown here. Changed material of the main text is copied here and shown in red.

Reviewer #1 (Remarks to the Author):

Electron ptychography has delivered record-breaking spatial resolution in numerous materials systems and under challenging experimental conditions (low dose, low-voltage, without Cs-corrector, thick specimen, etc.). Recently, the application of ptychography to reveal lattice-vibration-related features has been explored by several groups, which uncovered unique phenomena inaccessible by other imaging techniques, unleashing a new development thread of computational imaging. In this manuscript, the authors demonstrated that ptychographic reconstructions with multiple states of the object can deliver information about the correlated lattice vibrations. To my best knowledge, it is the first report of ptychographic reconstruction with mixed object states at atomic resolution. The manuscript is well organized and can be published with the following issues addressed.

> We would like to thank the reviewer for their thoughtful and constructive feedback. We address each point below.

1. The authors are encouraged to make the code, key raw datasets, and scripts for analysis public.

> We appreciate the suggestion. In response, we have uploaded both the ptychographic reconstruction code and the simulated and experimental 4D-STEM datasets to the Code Ocean platform. They can be accessed and downloaded via the following DOI: https://doi.org/10.24433/CO.7393167.v2. This link is now cited in the revised "Data Availability" and "Code Availability" sections of the manuscript. We further uploaded the ptychographic code to a public GitHub repository https://github.com/Anton-Gladyshev/pypty.

2. The authors should cite previous work studying the lattice-vibration-related phenomena using ptychography. For example, the recent one by Huang's group (Atom-by-atom imaging of moiré phasons with electron ptychography).

> We thank the reviewer for this important suggestion. We have cited the recommended work on page 1 of the main text and clarified its relationship to our contribution.

Ptychography has proven itself as a powerful tool for imaging of thin samples [5, 13]. Recently it was shown that adopting a multislice formalism makes it possible to resolve specimen features as fine as the blurring due to the vibrations of atoms [14]. This publication has had greatly impacted the electron microscopy community and triggered a rapid rise in popularity of ptychography. It was demonstrated that spatially non-uniform vibrational modes can be fitted to the atomic shapes produced via ptychography [15]. However, fully surpassing this limit of atomic vibrations was not possible, since a coherent specimen model as depicted in Fig. 1a cannot describe the formation of an incoherent diffraction pattern of the type schematically shown in Fig. 1c.

3. The authors used mix-state probes in the reconstruction. So please provide all the probes in the supplementary file. In addition, are the probes updated in the second stage of the reconstruction of Fig. 2e? Please make it clear how the probes are fitted and explain why fitted probes are necessary in the second stage, while not in the first stage.

> We agree this requires clarification. Multiple probe modes were used only in the later stages of reconstruction to reduce computational cost. This is now clearly stated on page 3 of the Supplementary Materials. Figures S6 and S7 have been added, showing probe modes recovered from the silicon and hBN datasets, respectively.

The loss derivative and corresponding update-vectors, are computed via Wirtinger calculus [15] a limited-memory BFGS (l-BFGS) algorithm [15, 16]. For the simulated silicon data no additional regularization was employed and an initial guess for the object was created using a uniform prior. The reconstruction shown in Fig. 2e of the main text went in two stages: 1) An initial reconstruction with a single object mode and 9 probe modes initialized using Hermite polynomials [7]; 2) A final reconstruction using a probe fitted during the first stage with 20 object modes. This scheme allows to apply a constraint on the recovered correlations

by absorbing as much collective motion of the beam or sample during the first stage and recover the non-uniform vibrations during the second one. The real-space wavefront of probe modes recovered from the silicon dataset with partial spatial coherence are shown in Fig. S6.

The experimental hBN dataset was padded with zeros in reciprocal space up to a shape of 732 ×732 pixels resulting in a pixel size in the ptychographic reconstruction of 10.89 pm. We further applied a 2/3 frequency cutoff to all intermediate waves (i.e. between all slices) to prevent aliasing artifacts [9]. The reconstruction went in four stages: 1) initial reconstruction with a single probe mode, single object mode and 30 object slices. This allowed us to estimate the aberrations of the beam. Further, cross-correlating the slices with their next neighbors allowed us to determine a minuscule difference between the zone axis of the crystal and the beam propagation direction. We used this value and adopted a tilted propagator [6] as defined in S5 without an additional refinement of the angle for all subsequent steps. A position-averaged diffraction pattern demonstrating the amount of misorientation is shown in Figure Fig. S8. Multiple probe modes could have been used during this stage. However, for the sake of reconstruction speed, they were introduced in the next stage. 2) reconstruction with 10 probe modes and a single object mode without any regularization using the angle fitted during stage 1; 3) The five dominant probe modes out of ten total modes recovered during the second stage were selected and 10 object modes were generated by adding a uniform prior (noise) to the object recovered at stage 2; 4) After the object at stage 3 was free from noise we added at the beginning, we applied regularization to the object, in particular, an l1-regularization [3] to the absorption potential enforcing the amplitude of the retrieved transmission function to be closer to 1, and also a missing-wedge regularization [6,7] which dampens high kz Fourier-components at small kx and ky. The probe modes recovered after the final stage of a reconstruction from the experimental hBN dataset are shown in Fig. S7.

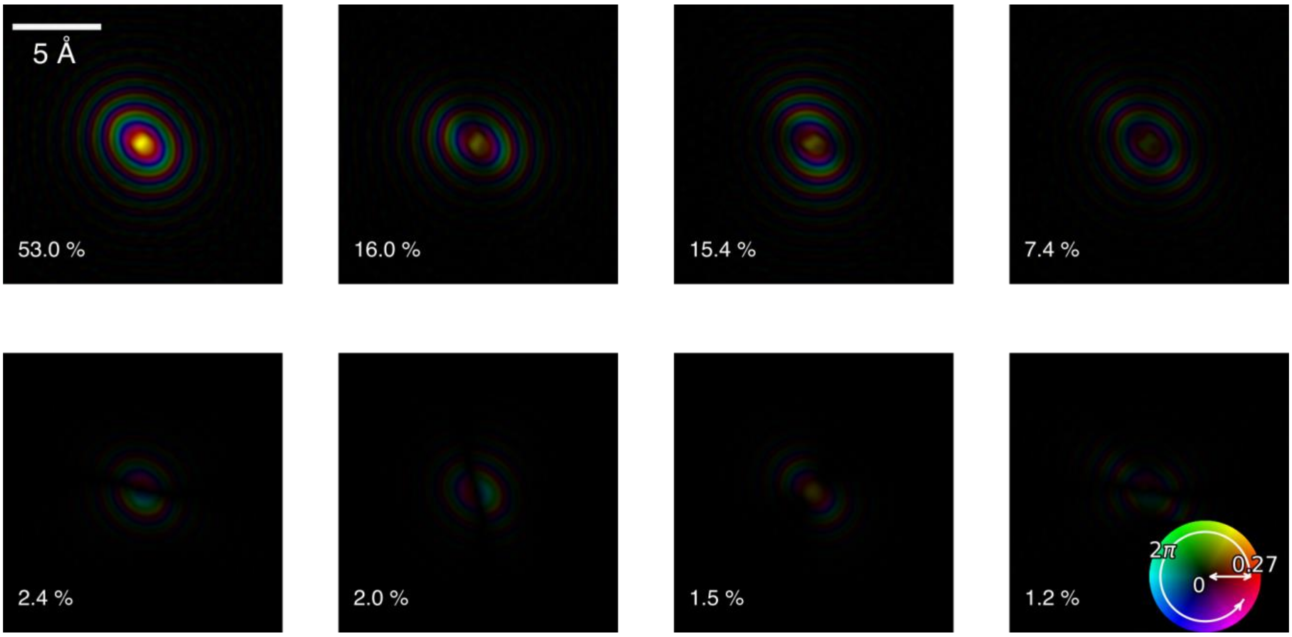

Fig. S6 | Real-space wavefronts of probe modes reconstructed from a simulated 4D-STEM dataset of a symmetric Σ9 grain boundary in silicon, including the effect of partial spatial coherence. Eight dominant out of the nine modes are shown using a complex colormap, where color represents the phase and brightness denotes the amplitude. The percentage of the total intensity captured by each mode is reported in the lower left corner of each image.

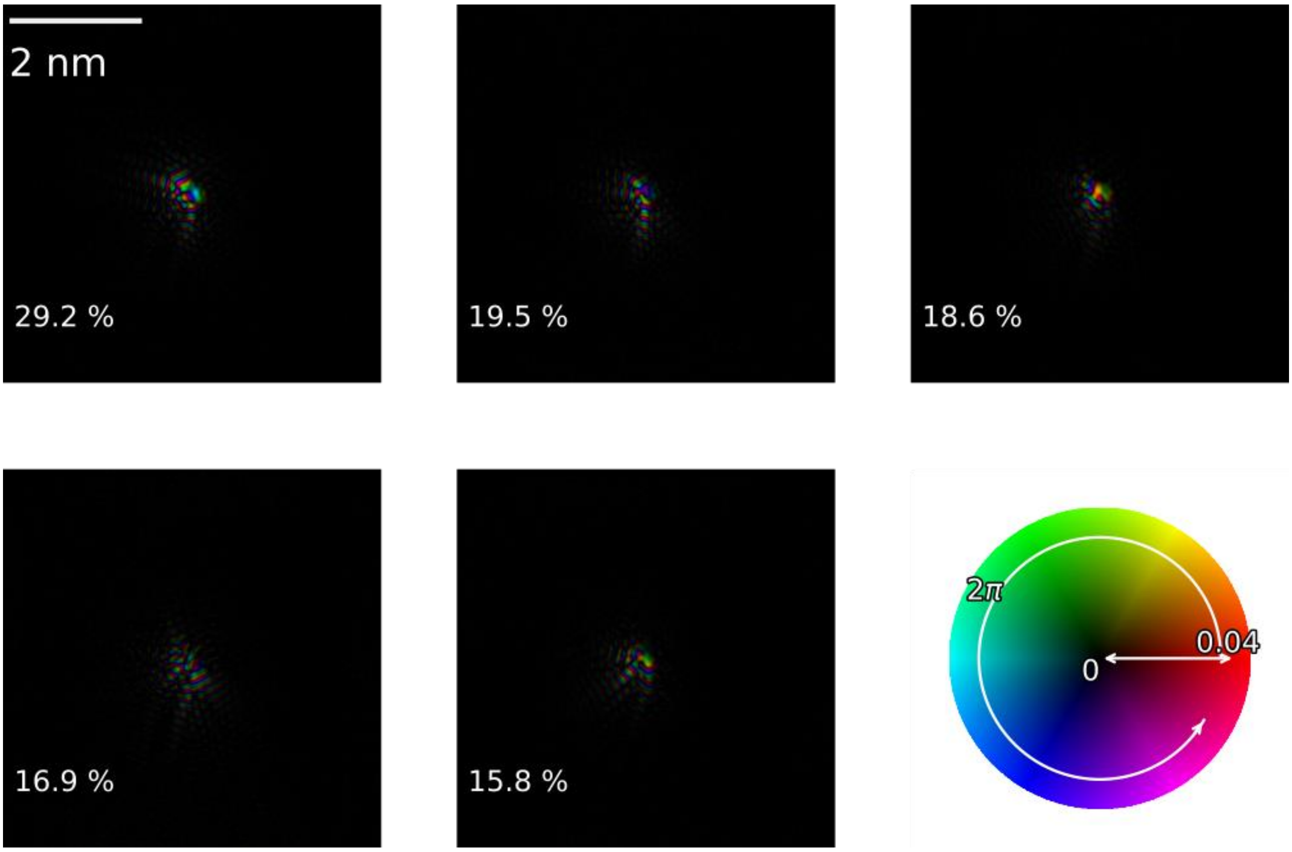

Fig. S7 | Real-space wavefronts of probe modes reconstructed from an experimental 4D-STEM dataset of a twisted hBN bicrystal. All five reconstructed modes are shown using a complex colormap, where color represents the phase and brightness denotes the amplitude. The percentage of the total intensity captured by each mode is reported in the lower left corner of each image.

4. “Further, cross-correlating the slices with their next neighbors allowed us to determine a minuscule difference between the zone axis of the crystal and the beam propagation direction. We used this value and adopted a tilted propagator [6] as defined in S5 without an additional refinement of the angle for all subsequent steps.”
First, the authors should provide PACBED showing the degree of off-zone and quantify the deviation. Second, the authors should evaluate how the deviation from the zone axis affects the results.

We appreciate the reviewer’s point. Position averaged convergent beam electron diffraction (PACBED) pattern is useful for single crystals, where all one needs is to refine its thickness and tilt. Here we would have to refine two thicknesses and a misorientation angle and possibly two different tilts. Fitting the tilt with ptychographic slices makes it possible to avoid these issues. Further, since our slice thickness was set to 0.48 nm, i.e. 1.5 times smaller than the unit cell, we do not expect to see any effects from misorientation despite a shift of atomic column positions in adjacent slices.

We added a Figure S8 showing PACBED and an amount of misorientation to the supplementary files.

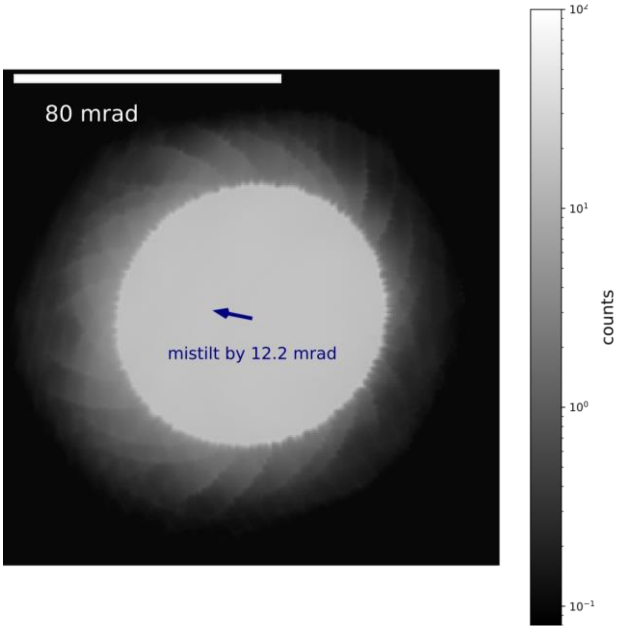

Fig. S8 | Position-averaged convergent-beam electron diffraction pattern (PACBED) obtained from a 4D- STEM dataset of a twisted hBN bicrystal. The mistilt angle of 12.2 mrad, manifested by the shift of the scattered discs and calculated from a ptychographic reconstruction, is indicated by the blue arrow.

5. “Moreover, using only the masses of the atomic species and the temperature as input, we obtain average frequencies of 10.8±0.1, 13.6±0.6, 18.0±0.2, 25.5±1.5 THz for the longitudinal and transversal acoustic and optic phonons, respectively, in agreement with inelastic neutron scattering, albeit from just a few nm3 volume.”
As the beam irradiation can heat the sample, how does the temperature affect the results?

We added a statement in the main text (page 7) explaining that beam-induced heating is minor, typically raising the temperature by only tens of Kelvin, and cannot account for the 1.5-fold discrepancy in phonon energies. Our analysis shows that underestimation of vibration amplitudes, not temperature, is the main factor affecting extracted frequencies.

We obtain 10.7 THz, 14.1 THz, 17.7 THz and 24.0 THz for transverse acoustic, longitudinal acoustic, transverse optical, longitudinal optical branches in the upper lattice. For the bottom lattice we get 10.8 THz, 13.0 THz, 18.2 THz, and 27.0 THz for the same four branches. The reported theoretical values are approximately 1.5 times higher: 14.3 THz, 22.9 THz, 39.5 THz and 39.6 THz [40]. The scaling of energies calculated via CAVIAR is affected by both assumed temperature as well as extracted amplitude of atomic vibrations. Although an electron beam heats the sample during data collection, this effect is negligible, as the temperature raises only by up to a few tens of Kelvin, which is not enough to observe a noticeable change in energy. The vibration amplitude is a far more important factor, and our simulations show that CAVIAR can underestimate it. Still, CAVIAR makes it possible to place frequencies of two acoustic and two optical branches in the correct range for a complementary Vibrational STEM-EELS measurements [25, 41]. Further, unlike EELS, mixed-object ptychography yields directions of correlated vibrations at the level of a single atom.

6. “Fig. 3c and clearly indicates an information transfer within the full convergence angle of the beam (80 mrad) [14, 21].”
In principle, the resolution should be somewhat improved as the sample incoherence is addressed. Why is the resolution still limited to 2 alpha?

We would like to point out that this sentence referred to the depth resolution and not to the in-plane resolution. The exact bound for achievable depth resolution is an open topic and the most detailed explanation is given by Z. Dong et.al. in [10.1038/s41467-025-56499-1]. Although the collection

angle plays a role in a theoretical model, the depth resolution in published multislice ptychographic reconstructions up to our best knowledge, as in our case, appears to be bounded by the convergence angle. Still, even if the depth resolution would exceed the enclosed cone, the picometer scale atomic vibrations should not play a significant role for object slices with an Ångstrom-scale thickness. We would like to point out that the in-plane resolution achieved of 47.8 pm exceeds 61 pm - the Abbe-resolution limit of 2 alpha. where the picometer-scale atomic vibrations become more relevant. At the initial phase of this study we aimed to observe an improvement of the in-plane resolution. But we did not observe a further improvement in resolution after using mixed object states. This might be explained by the fact the atomic vibrations do not necessarily correspond to highly periodic features directly interpretable via fast Fourier transform.

We reformulated the sentence on page 5 of main text.

The corresponding power spectrum is shown in Fig. 3c and clearly indicates a missing wedge of three dimensional information [14]. Albeit the in-plane frequencies show an information transfer reaching beyond the double aperture limit, the depth spatial frequencies are still confined in a full convergence angle, in agreement with previously published multislice ptychographic reconstructions [22]. The first two rings of hBN reflections correspond to a depth resolution of approximately 2 nm.

7. “The reconstructed transmission function included 10 object states and 30 slices with a spacing of 0.48 nm.”
As there is no ground truth for experimental datasets, the authors should test the number of object states.

We updated our ptychographic reconstruction code and implemented features that make it possible to fit more object states into GPU memory. The key improvement is an ability to store as much data as possible, such as gradients and the history of previous updates, on the CPU, while sending only the data immediately needed for multislice computation to the GPU. Although this additional data transfer slightly slows down the reconstruction, we were able to successfully perform a reconstruction from the experimental hBN data using 5 probe states and twice as many object states (20) as in the original implementation. The extracted correlations do not show a significant improvement compared to the 10-object-state reconstruction initially presented in the paper. These findings are summarized in the newly added section Benchmarking Temporal Resolution and Recovery of Interatomic Vibrations in supplementary materials.

The CAVIAR reconstructions from a simulated 4D-STEM dataset of a silicon grain boundary, shown in Fig.2 of the main text, demonstrate that while the directions of atomic vibrations can be retrieved with relatively little
information loss, the strength of the interatomic correlations is significantly underestimated. To further quantify this effect, we performed additional calculations using the raw molecular dynamics snapshots that were used to generate the 4D-STEM datasets. The initial frames were sampled with a 1 fs step [17]. Gaussian blurring was sequentially applied along the time axis with various standard deviations, effectively degrading the time resolution, and the resulting correlations were compared with unblurred data. The curves showing the response of the length similarity metric (LSM) and the cosine similarity metric (CSM), defined in Eqs. (3) and (4) of the main text, respectively, to time resolution are presented in Fig. S3. As the time resolution decreases, i.e., with increasing standard deviation, the LSM values drop exponentially, whereas the CSM curve resembles an arccotangent function, decreasing much more slowly. The plots show that a standard deviation of 0.67 fs yields an LSM value of 0.56 and a CSM value of 0.95, closely matching the benchmarks achieved by the CAVIAR reconstruction presented in Fig. 2e of the main text. While the origin of the time resolution still has to be investigated, it might be linked to the ratio between the detector pixel size, i.e. Fourier space sampling, and the size of features in the diffraction pattern that are unique for a particular set of interatomic correlations [18].

It has been shown that, for simulations of electron diffraction patterns, thermal diffuse scattering effects can be accounted for by using a few tens of atomic configurations, i.e. object states [19]. To benchmark the response of the CAVIAR reconstruction to the number of object states used, we performed an additional ptychographic reconstruction from the experimental 4D-STEM dataset of a twisted hBN bicrystal using 20 object states and 5 probe states. The initial guess for the object was created by stacking two copies of the result obtained from the 10-state reconstruction and adding a small amount of uniform prior to introduce initial variation across the states. Upon convergence, the interatomic correlations as well as the phonon dispersion curves were extracted via CAVIAR. The results of the 20-state reconstruction are presented in Fig. S5, using a similar style to that used for the 10-state reconstruction in Fig. S4. While the entries of the Green's tensor appear slightly less noisy in the 20-state reconstruction, the corresponding phonon dispersion curves do not show significant changes. Furthermore, the average energies of four branches remain approximately 1.5 times lower than reported theoretical values [20] and show no improvement. Taken together with the time-resolution dependence of the LSM metric, these results suggest that time blurring, rather than the number of object states, is the dominant factor limiting the reconstruction quality. Moreover, in case of the experimental data the extracted interatomic correlations may be further degraded by Poisson noise present in the diffraction patterns.

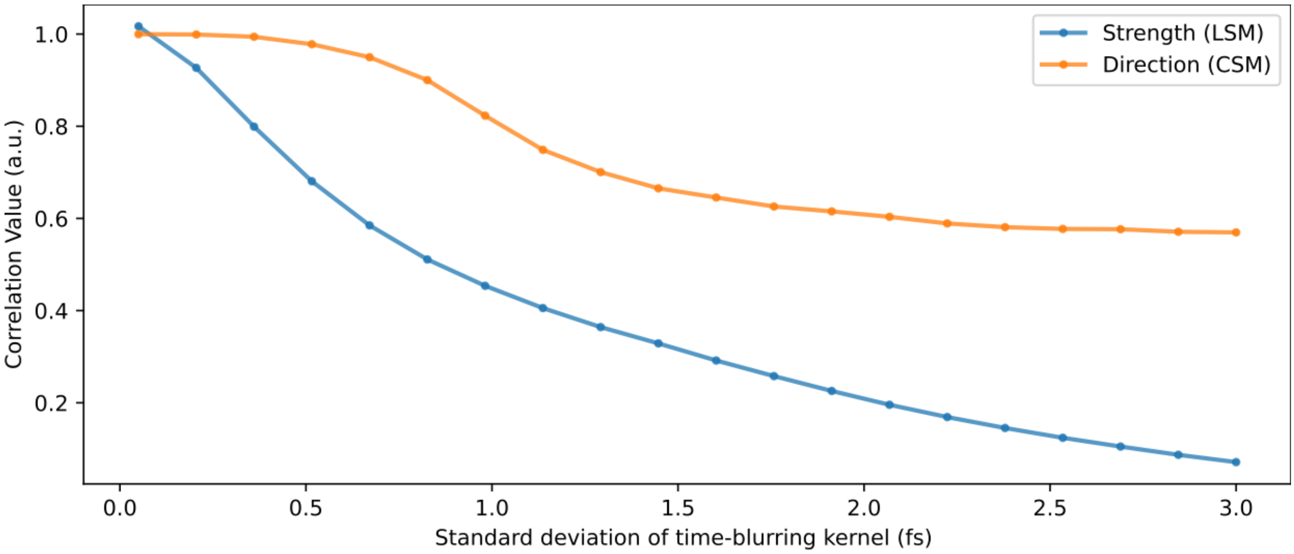

Fig. S3 | Dependence of benchmarking metrics for strength (LSM) and direction (CSM) of reconstructed atomic vibrations on time resolution. The orange and blue curves illustrate the effect of applying Gaussian blurring along the time axis to molecular dynamics snapshots of a symmetric Σ9 grain boundary in silicon, showing how temporal smoothing alters the extracted strength and direction of atomic vibrations compared to unblurred data with 1 fs time sampling. The LSM and CSM metrics are defined in equations (3) and (4) of the main text, respectively.

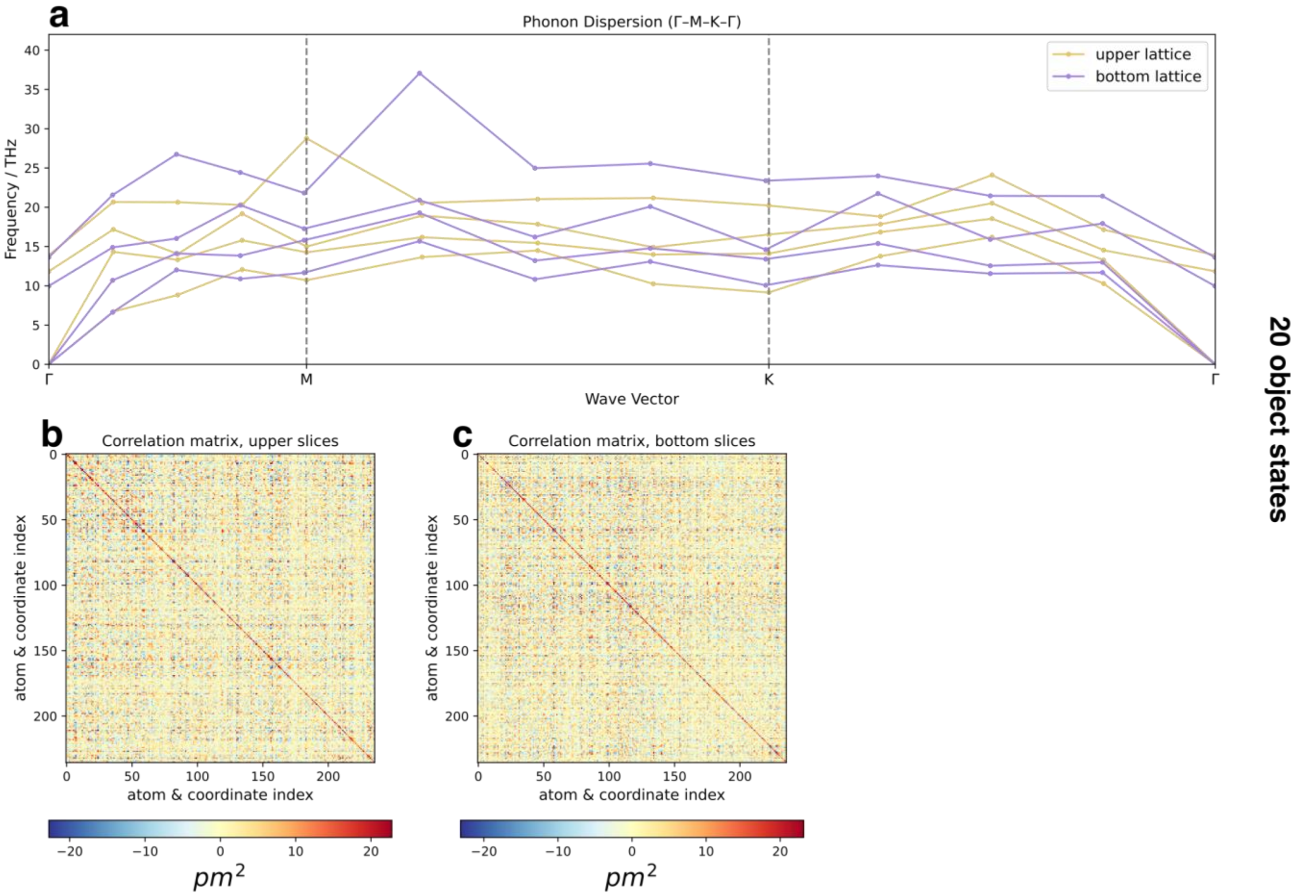

Fig. S5 | Interatomic correlations (Green's tensors) recovered from experimental 20-state mixed-object ptychographic reconstruction of approximately 15 nm thick bulk hBN crystal. a, Phonon dispersion curves extracted from the reconstructed atomic displacement correlations using the lattice Green's tensor method from an experimental bulk hBN dataset. Yellow curves show the dispersion from the upper hBN lattice layer, and purple curves the one from the lower layer. Both are computed along the Γ-M–K-Γ direction of the Brillouin zone. In the upper lattice the average frequencies along the Γ−K−M−Γ path are 11.5 THz, 15.1 THz, 17.0 THz and 21.2 THz for transverse acoustic, longitudinal acoustic, transverse optical, longitudinal optical branches in the upper lattice. For the bottom lattice we get 11.5 THz, 14.2 THz, 17.8 THz, and 24.8 THz for the same four branches. Panels b and c show average correlation matrices extracted from a z-range z = 1.9 nm...5.7 (top lattice) and z= 8.6 nm...12.5 nm (bottom lattice), respectively.

Reviewer #2 (Remarks to the Author):

The paper by Gladyshev et al reports on a further new development in the filed of electron ptychography in which the authors extend the previously reported 3D multislice approach to a 4D mixed object multislice ptychography which provide information on lattice vibrations. As such the paper should certainly be published in nature Communications.

I have some suggestions / questions that the authors might wish to address but these are in the spirit of constructive comments and none of them should preclude publication.

We thank the reviewer for their supportive and insightful comments. We respond to each point below.

A. The comparison with vibrational EELS is, in my view the most powerful point in the paper but it is somewhat buried late in the introduction. Maybe a mention in the abstract would be helpful. It might also be useful to have an indication of the momentum / spatial limits of the new method and a comparison of these to the limits in Vibrational EELS- an initial crude calculation suggests that access to the phonon spectra should be possible experimentally at close to atomic resolution in x-y and this is highly significant.

We agree and revised the abstract to emphasize CAVIAR's potential as a complementary technique to vibrational EELS.

This ability to spatially resolve correlated atomic motion distinguishes CAVIAR and positions it as a complementary tool to vibrational electron energy loss spectroscopy for exploring atom dynamics at the finest scale with the potential to be instrumental in the development of phononic devices, in studying phonon-based decoherence in quantum systems, or other emerging phonon-based applications.

B. In Figure 2 (at low resolution) it seems that CAVIAR with correlated vibrations but without FSC gives similar results for the self correlations but that the advantage of including FSC comes through in the correlation with the reference atom and in better fidelity of the angles recovered. Moreover in both cases as the authors note the magnitude of the correlations in all cases is lower that that of the ground truth. The authors suggest that this latter effect is due to a resolution limit in the reconstruction. It might be helpful to compare the reconstructions with a de-resolved (blurred) version of the ground truth. I also missed the temperature of the MD simulations and possible a slight adjustment to this would improve the match in the magnitudes. This would also give an indication of the likely precision recast as an error in the temperature.

We would like to thank the reviewer for this helpful suggestion. Although a blurring in the x-y plane will not be sufficient to mimic the degradation of the results since the center of mass of the atomic columns will not change, this comment encouraged us to test a blurring along the time axis. Limiting the time-resolution of the molecular dynamics frames makes it possible to reproduce benchmarking values similar to the ones obtained via CAVIAR reconstructions. We summarised our findings in a newly added section in the supplementary materials.

The CAVIAR reconstructions from a simulated 4D-STEM dataset of a silicon grain boundary, shown in Fig.2 of the main text, demonstrate that while the directions of atomic vibrations can be retrieved with relatively little information loss, the strength of the interatomic correlations is significantly underestimated. To further quantify this effect, we performed additional calculations using the raw molecular dynamics snapshots that were used to generate the 4D-STEM datasets. The initial frames were sampled with a 1 fs step [17]. Gaussian blurring was sequentially applied along the time axis with various standard deviations, effectively degrading the time resolution, and the resulting correlations were compared with unblurred data. The curves showing the response of the length similarity metric (LSM) and the cosine similarity metric (CSM), defined in Eqs. (3) and (4) of the main text, respectively, to time resolution are presented in Fig. S3. As the time resolution decreases, i.e., with increasing standard deviation, the LSM values drop exponentially,

whereas the CSM curve resembles an arccotangent function, decreasing much more slowly. The plots show that a standard deviation of 0.67 fs yields an LSM value of 0.56 and a CSM value of 0.95, closely matching the benchmarks achieved by the CAVIAR reconstruction presented in Fig. 2e of the main text. While the origin of the time resolution still has to be investigated, it might be linked to the ratio between the detector pixel size, i.e. Fourier space sampling, and the size of features in the diffraction pattern that are unique for a particular set of interatomic correlations [18].

It has been shown that, for simulations of electron diffraction patterns, thermal diffuse scattering effects can be accounted for by using a few tens of atomic configurations, i.e. object states [19]. To benchmark the response of the CAVIAR reconstruction to the number of object states used, we performed an additional ptychographic reconstruction from the experimental 4D-STEM dataset of a twisted hBN bicrystal using 20 object states and 5 probe states. The initial guess for the object was created by stacking two copies of the result obtained from the 10-state reconstruction and adding a small amount of uniform prior to introduce initial variation across the states. Upon convergence, the interatomic correlations as well as the phonon dispersion curves were extracted via CAVIAR. The results of the 20-state reconstruction are presented in Fig. S5, using a similar style to that used for the 10-state reconstruction in Fig. S4. While the entries of the Green's tensor appear slightly less noisy in the 20-state reconstruction, the corresponding phonon dispersion curves do not show significant changes. Furthermore, the average energies of four branches remain approximately 1.5 times lower than reported theoretical values [20] and show no improvement. Taken together with the time-resolution dependence of the LSM metric, these results suggest that time blurring, rather than the number of object states, is the dominant factor limiting the reconstruction quality. Moreover, in case of the experimental data the extracted interatomic correlations may be further degraded by Poisson noise present in the diffraction patterns.

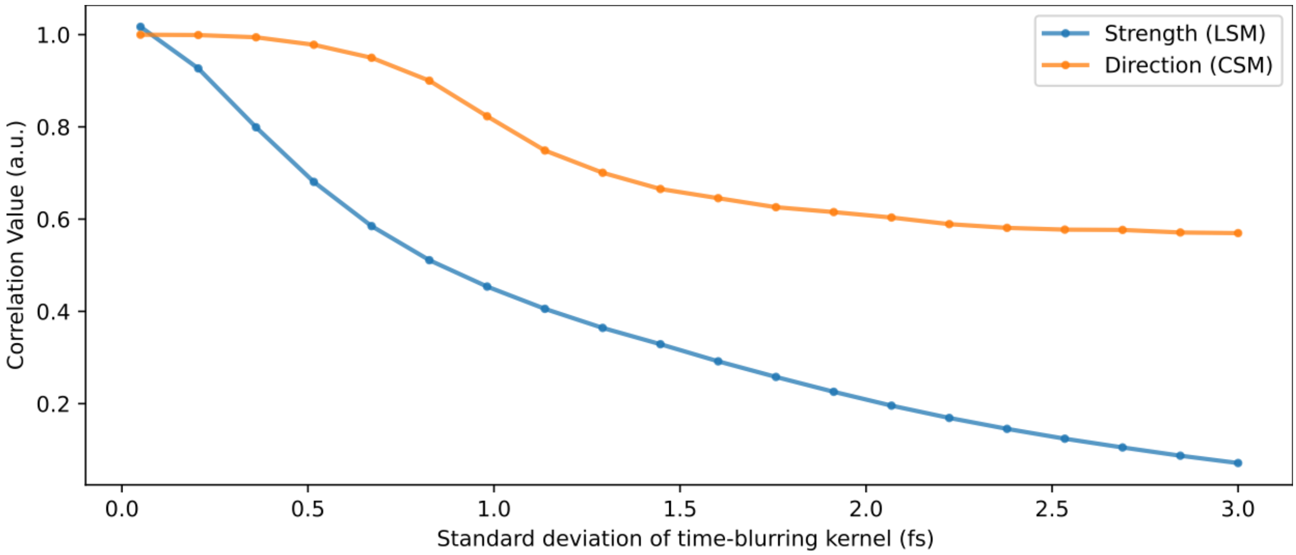

Fig. S3 | Dependence of benchmarking metrics for strength (LSM) and direction (CSM) of reconstructed atomic vibrations on time resolution. The orange and blue curves illustrate the effect of applying Gaussian blurring along the time axis to molecular dynamics snapshots of a symmetric Σ9 grain boundary in silicon, showing how temporal smoothing alters the extracted strength and direction of atomic vibrations compared to unblurred data with 1 fs time sampling. The LSM and CSM metrics are defined in equations (3) and (4) of the main text, respectively.

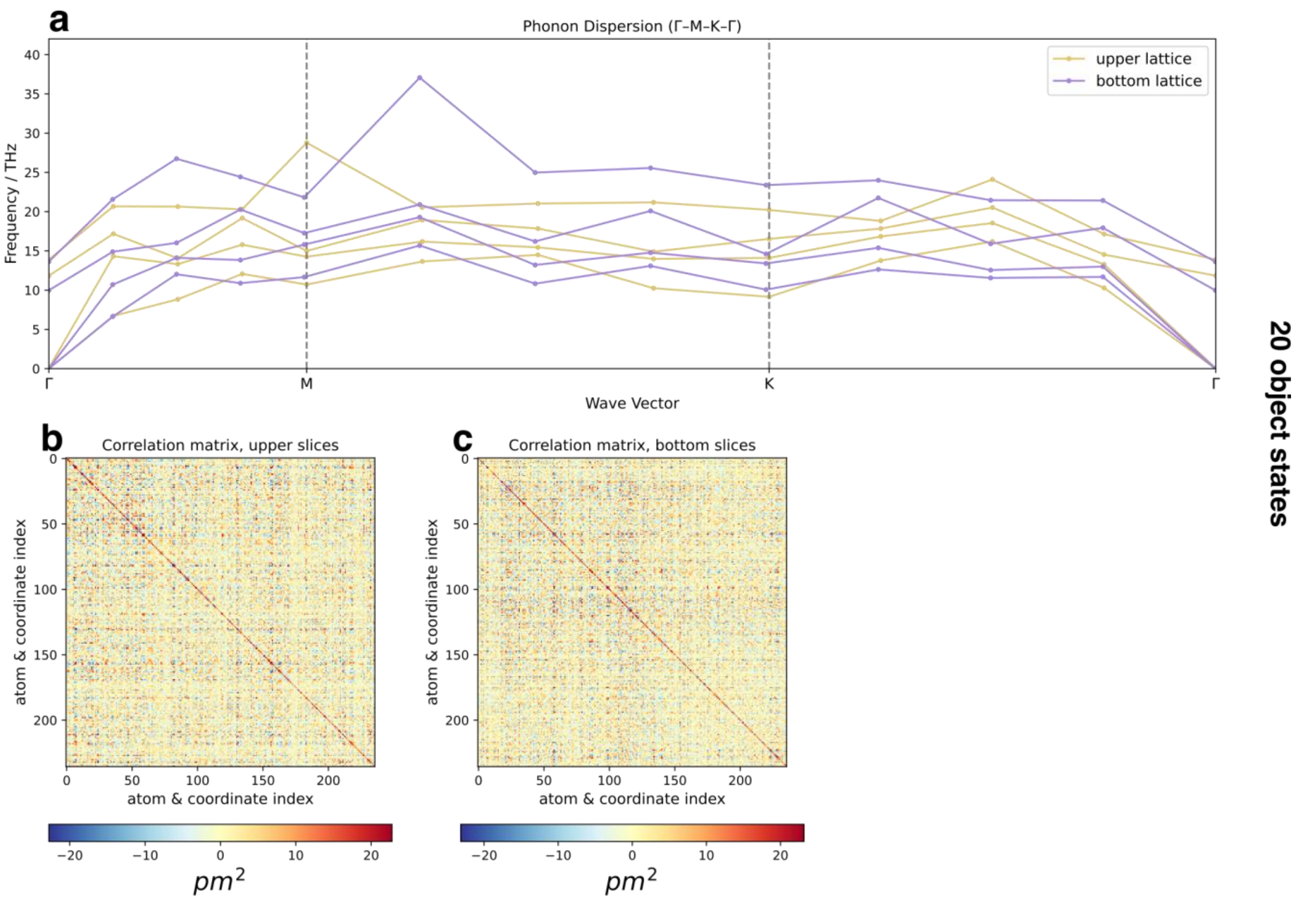

Fig. S5 | Interatomic correlations (Green's tensors) recovered from experimental 20-state mixed-object ptychographic reconstruction of approximately 15 nm thick bulk hBN crystal. a, Phonon dispersion curves extracted from the reconstructed atomic displacement correlations using the lattice Green's tensor method from an experimental bulk hBN dataset. Yellow curves show the dispersion from the upper hBN lattice layer, and purple curves the one from the lower layer. Both are computed along the Γ-M–K-Γ direction of the Brillouin zone. In the upper lattice the average frequencies along the Γ−K−M−Γ path are 11.5 THz, 15.1 THz, 17.0 THz and 21.2 THz for transverse acoustic, longitudinal acoustic, transverse optical, longitudinal optical branches in the upper lattice. For the bottom lattice we get 11.5 THz, 14.2 THz, 17.8 THz, and 24.8 THz for the same four branches. Panels b and c show average correlation matrices extracted from a z-range z = 1.9 nm...5.7 (top lattice) and z= 8.6 nm...12.5 nm (bottom lattice), respectively.

C. On p6 (in my copy) it is stated that 10 object states may not be sufficient and it wold help to have an approximate indication of the number required to accurately model the TDS and Debye Waller factor.

> For multislice simulations, it has been shown that simulated diffraction patterns converge after a few tens of object states (see, for example, Figure 12 on page 27 of: Koch, C. T., Determination of core structure periodicity and point defect density along dislocations. Arizona State University, 2002). In the context of iterative mixed-object ptychography, we have now addressed this question in the supplementary section "Benchmarking Temporal Resolution and Recovery of Interatomic Vibrations", which is pasted above in our response to point "B". While reconstructions using 20 object states yielded slightly less noisy correlation maps compared to 10 states, we did not observe significant improvement in the extracted phonon energies. These findings suggest that the temporal resolution of the data, rather than the number of object states used in the reconstruction, is the dominant factor limiting the accuracy of vibrational recovery.

D. Finally the authors correctly point out on p4 that the current depth resolution in STEM is insufficient to recover a full 3D reconstruction. It would be useful to have firstly an indication of the resolution limit required and the electron optical correction needed to achieve this. I suspect that it may not be achievable

with current correctors for hard materials that require high depth resolution but there may be interesting applications to soft materials at lower resolution.

> We thank the reviewer for inspiring comment. A new supplementary section titled "Remarks on a Full 3D Reconstruction" has been added. It discusses angular requirements for full 3D reconstruction and current limitations in corrector technology. We conclude that while challenging, achieving this may be feasible with large convergence angles and novel detector configurations in future setups.

A truly full 3D reconstruction from data recorded for just a single sample orientation is not possible, as the lowest spatial frequencies along the beam direction are lost due to the finite convergence angle of the illumination [21, 22]. However, although very challenging experimentally, the determination of 3D atomic positions from a single-tilt 4D-STEM dataset may still be theoretically possible. Unlike in projection imaging, the minimal realistic interatomic distance in three dimensions is around 2 Å. Achieving such depth resolution for median in-plane spatial frequencies between 1/2 $Å^{-1}$ and 1 $Å^{-1}$, corresponding to in-plane resolutions of 2 Å and 1 Å, respectively, requires convergence semi-angles in the range between arctan(0.5/2) and arctan(0.5/0.1), approximately 245 and 464 mrad. While angles as large as 0.464 rad are likely unrealistic experimentally, the lower bound of 245 mrad may become feasible in the future. While aberration correctors are currently able to reduce aberrations up to approx. 50 mrad, they increase aberrations beyond that. It may thus be preferential to perform such experiments in an uncorrected TEM. Sub-A ptychographic reconstructions using such instrument has recently been demonstrated [23]. From our perspective, a far more critical limitation is the number of pixels in hybrid-pixel direct electron detectors, as well as the acceptance angles of electromagnetic lenses and apertures. While there are no fundamental physical constraints preventing such a configuration of an electron microscope, the required development would likely be technically challenging, and we are not aware of any ongoing engineering efforts aimed at extending this angular range.

Finally to reiterate the above are intended as helpful suggestions in part to satisfy my interest and are not intended to prevent publication of this excellent work

Reviewer #2 (Remarks on code availability):

The code provided is sufficient to allow others to reproduce that data reported.

Additional changes in the manuscript

1. We identified an error in the code used to compute the length similarity metric (LSM) values presented in Figure 2 of the main text. The figure has been updated with the corrected values. Additionally, the text on page 5 was revised to replace "25%" with "two times" to reflect the corrected interpretation. We sincerely apologize to the editors and reviewers for any inconvenience this may have caused.

We updated Figure 2 and text on page 5 of the main text.

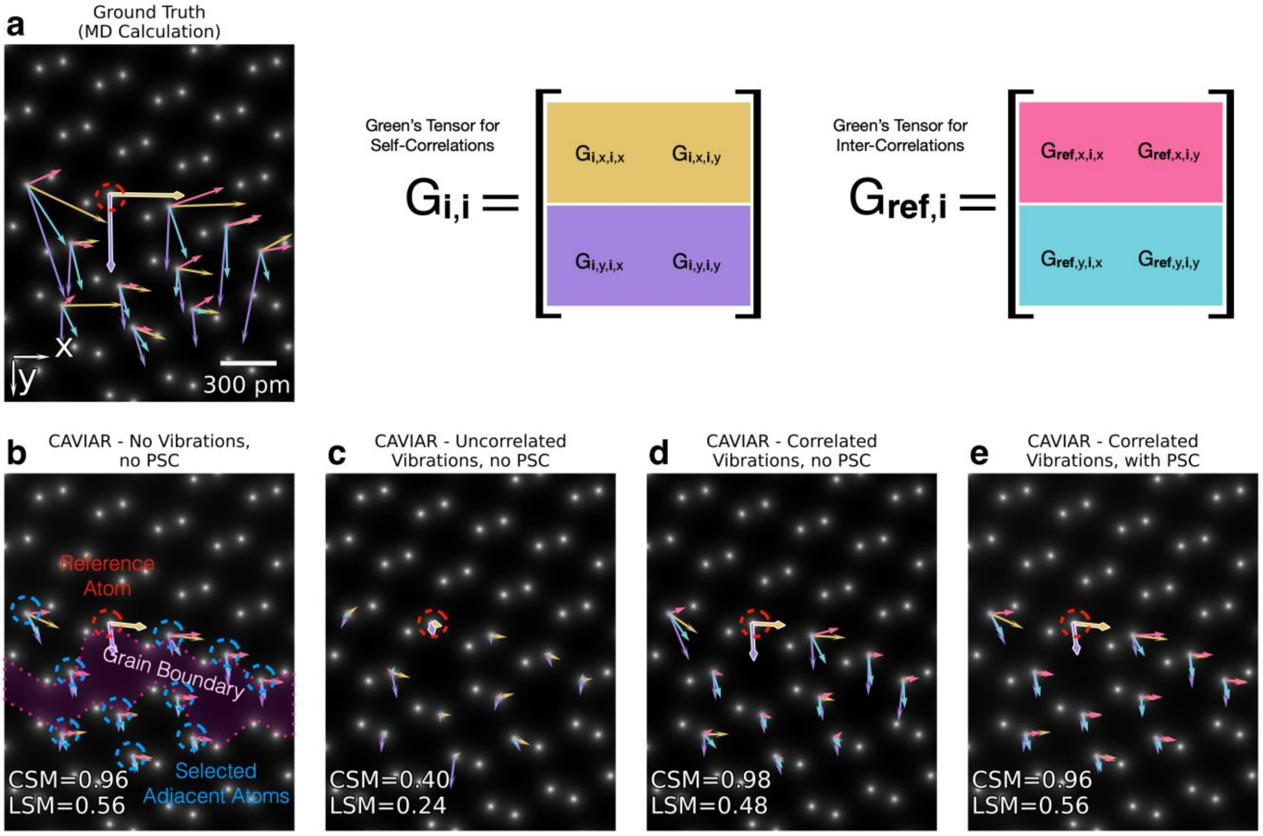

Fig. 2 | Ptychographic reconstructions from realistically simulated data for various kinds of atomic motion and partial spatial coherence (PSC). a, Ground truth phase image from an MD simulation of a Σ9 grain boundary in silicon with arrows indicating various components of the Green's tensor defined in eq. 2. A randomly picked reference atom is marked by a red circle in a – e, its selected neighbors and the grain boundary are shown by a blue and magenta dashed lines in b, respectively. Colors of arrows show different correlation types: yellow/purple (self-correlations), pink/light blue (correlation with reference atom), two 2 ×2 blocks of the full Green's tensor are shown schematically in the middle of the top row using the same colors. b – e, Reconstructed phases and correlation matrices obtained from data simulated using different levels of approximation: b Perfectly coherent dataset, no PSC — no correlations appear to have been reconstructed; c Uncorrelated vibrations (Einstein model), no PSC — correlations between neighbors largely vanish; d Correlated vibrations, no PSC — principal directions of correlations match the ground truth; e Correlated vibrations, with PSC — the recovered correlations match the ground truth and the offset created by PSC is efficiently mitigated by multiple probe modes; In the bottom of images b – e we report values of length-similarity (LSM) and cosine-similarity (CSM) metrics (cf. eq. 3 and 4 in the main text) between MD ground truth and retrieved correlation vectors. All reconstructions used 4 object slices, 20 object states. Reconstructions in b – d were done with a single probe mode. In the reconstruction e 9 probe modes were used.

2. We fixed terminology: changed "transversal" to "transverse" in the abstract.

3. We changed “prove another useful tool” to “Prove useful as another tool” on page 2 of the main text.

4. We changed “Indicating the partial spatial coherence” to “Allowing for the partial spatial coherence” on page 3 of the main text.

5. Minor grammatical and spelling corrections have been made to the supplementary materials.

6. Added Sections "Data Availability","Code Availability" and "Competing Interests" to the main text.

Data Availability

The simulated and experimental 4D-STEM datasets, together with the corresponding ptychographic reconstructions presented in this study, have been deposited on the Code Ocean platform under the accession code https://doi.org/10.24433/CO.7393167.v2.

Code Availability

The custom-developed ptychographic reconstruction software and analysis tools used in this study are openly available on the Code Ocean platform at https://doi.org/10.24433/CO.7393167.v2. The source code for the ptychographic reconstruction engine is also hosted on GitHub at https://github.com/Anton-Gladyshev/pypty.

Competing Interests

The authors declare no competing interests.
